# miR-199a-3p/5p regulate tumorgenesis via targeting Rheb in non-small cell lung cancer

**DOI:** 10.7150/ijbs.70312

**Published:** 2022-06-27

**Authors:** Xiaomin Liu, Xianyi Wang, Binshu Chai, Zong Wu, Zhitao Gu, Heng Zou, Hui Zhang, Yanli Li, Qiangling Sun, Wentao Fang, Zhongliang Ma

**Affiliations:** 1 Lab for Noncoding RNA & Cancer, School of Life Sciences, Shanghai University, Shanghai 200444, China.; 2 Department of Thoracic Surgery, Thoracic Cancer Institute, Shanghai Chest Hospital, Jiaotong University Medical School,Shanghai 200030, China.

**Keywords:** NSCLC, miR-199a-3p/5p, Rheb, tumor suppressor, tumor resistance

## Abstract

Lung cancer is one of the deadliest cancers, in which non-small cell lung cancer (NSCLC) accounting for 85% and has a low survival rate of 5 years. Dysregulation of microRNAs (miRNAs) can participate in tumor regulation and many major diseases. In this study, we found that miR-199a-3p/5p were down-expressed in NSCLC tissue samples, cell lines, and the patient sample database. MiR-199a-3p/5p overexpression could significantly suppress cell proliferation, migration ability and promote apoptosis. Through software prediction, ras homolog enriched in brain (Rheb) was identified as a common target of miR-199a-3p and miR-199a-5p, which participated in regulating mTOR signaling pathway. The same effect of inhibiting NSCLC appeared after down-regulating the expression of Rheb. Furthermore, our findings revealed that miR-199a can significantly inhibit tumor growth and metastasis *in vivo*, which fully demonstrates that miR-199a plays a tumor suppressive role in NSCLC. In addition, miR-199a-3p/5p has been shown to enhance the sensitivity of gefitinib to EGFR-T790M in NSCLC. Collectively, these results prove that miR-199a-3p/5p can act as cancer suppressor genes to inhibit the mTOR signaling pathway by targeting Rheb, which in turn inhibits the regulatory process of NSCLC. Thus, to investigate the anti-cancer effect of pre-miR-199a/Rheb/mTOR axis in NSCLC, miR-199a-3p and miR-199a-5p have the potential to become an early diagnostic marker or therapeutic target for NSCLC.

## Introduction

In accordance with the latest world cancer report in 2022, lung cancer is one of the deadliest cancers with the highest mortality and morbidity rates in both men and women 1-3. Lung cancer is divided into small cell lung cancer (SCLC) accounting for 15% and non-small cell lung cancer (NSCLC) accounting for 85% 4. Because early stage lung cancer patients do not show obvious symptoms of the disease, about 40% of NSCLC patients are detected to have brain metastases during the progression of the disease 5. Coupled with the lack of effective measures for early diagnosis and prognosis of lung cancer patients, the five-year survival rate of NSCLC patients is not more than 15% 6, 7. Epidermal growth factor receptor (EGFR) is an essential driver gene in lung cancer 8. Studies have shown that EGFR overexpression or active mutations account for about 30% in lung cancer 9, and targeted EGFR therapy has become one of the most important treatment options for NSCLC 10. EGFR tyrosine kinase inhibitor (EGFR-TKI) is developing rapidly as a target drug for lung cancer 11. 50% of acquired resistance to EGFR-TKI is due to the presence of the T790M mutation, which increases the affinity of the ATP-binding site for ATP and inhibits the binding of TKI to this site, resulting in drug resistance for the patients 12-14. Therefore, it is clinically important to investigate the specific mechanisms in EGFR-TKIs resistance to alleviate drug resistance.

microRNA (miRNA) is a type of single-stranded non-coding RNA consisting of 18~24 nucleotides (nts) that participate in regulation of tumors and many major diseases 15-17. The involvement of miRNA can influence many aspects of lung cancer function 18. For instance, research shows that miRNA-9 can regulate the expression of IL-17A as an IL-17A mRNA antagonistic mediator 19. MiR-182-5p may act as an oncogene influence on NSCLC via regulating target genes such as HOXA9 20. MiRNA may exert oncogenic or suppressive influence on NSCLC through regulating target genes. In order to provide new perspectives for the diagnosis and treatment of lung cancer, the regulatory role of miRNAs needs more research.

Additionally, miRNA can participate in regulating the proliferation, apoptosis, metastasis, autophagy and other biological processes of tumor cells, via mediating the mTOR signal pathway. *Shen L.*et al study showed that miR-199a-3p inhibits the activation of mTOR signaling pathway and tumor proliferation in liver cancer, glioma and endometrial cancer 21. Among them, Rheb is an important signaling molecule upstream of the mTOR signaling pathway and belongs to the Ras/ Rap/Ral subfamily. The activation of Rheb will continue to activate the mTOR signaling pathway, which in turn affects the proliferation, apoptosis, drug resistance and autophagy of tumor cells 22, 23. In lung cancer, the regulatory role of miRNAs in mTOR signaling pathway is also a very significant research direction. Pre-miR-199a, a cancer-related miRNA, can be synthesized two mature miRNAs of miR-199a-3p and miR-199a-5 by cleavage from the 5' and 3' ends 24. Studies have revealed that two mature bodies of pre-miR-199a (miR-199a-3p and miR-199a-5p) play different roles in many tumors and have effects on the activation of multiple signaling pathways 25-27.* Wang Y.* et al research has shown that miR-199a-5p regulated the pathogenesis of ankylosing spondylitis via targeting Rheb 28. But the mechanism of miR-199a-3p and miR-199a-5p in lung cancer are not yet fully understood, so we investigate the role of both miR-199a-3p and miR-199a-5p.

In this study, it showed that miR-199a-3p and miR-199a-5p were down-expressed in NSCLC tissue samples, cell lines, and the patient sample database. When miR-199a-3p and miR-199a-5p are over-expressed in NSCLC cell lines, apoptosis was significantly increased, cell proliferation and migration ability were suppressed. Both *in vitro* and *in vivo*, miR-199a-3p/5p acted as an important tumor suppressor in NSCLC with Rheb as a novel target. MiR-199a-3p/5p could enhance the sensitivity of gefitinib to EGFR-T790M in NSCLC. MiR-199a-3p and miR-199a-5p have the potential to become an early diagnostic marker or therapeutic target for NSCLC.

## Materials and methods

### Lung cancer tissue samples

The tissue samples were obtained from Shanghai Chest Hospital, affiliated with Shanghai Jiao Tong University. The ethical approval was granted by the ethics committee of Shanghai Chest Hospital. The clinical tissue sample information is presented in Supplemental Table 1.

### Cell culture

HCC827, BEAS-2B and HEK293T cells were obtained from the Cell Bank, China Academy of Sciences (Shanghai, China). H1975, H1650, A549, H1299, and PC-9 cells were purchased from the ATCC (American Type Culture Collection, Manassas, VA, USA). The BEAS-2B cell line was isolated from normal human bronchial epithelium. A549, PC-9, and HEK293T cells were cultured in Dulbecco's modified Eagle's medium (DMEM, Corning Cellgro, Manassas USA) and BEAS-2B cells were cultured in LHC-9 medium. HCC827, H1975, H1650, and H1299 were cultured in either RPMI-1640 medium. All media were supplemented with 10% fetal bovine serum (FBS, HyClone Laboratories, Logan, UT, USA), an antibiotic cocktail of 100 U/mL penicillin, and 100 μg/mL streptomycin (Gibco). Cell culture was carried out at 37˚C in a 5% CO_2_ humidified environment.

### Cell transfection

MiR-199a-3p/5p mimic, NC mimic, miR-199a-3p/5p inhibitor, NC inhibitor, agomiR-199a-3p/5p and agomiR-NC were purchased from Ribobio (Guangzhou, China), which were then transiently transfected in A549, H1299, HEK293T and PC-9 cells using Lipofectamine 2000 (Invitrogen™ Life Technologies, New York, USA), according to the manufacturer's instructions. The same instructions were applied to the transfection of shRNA (shNC and shRheb). After 24 to 48 h post-transfection, cells were used for subsequent experiments including assays for cell proliferation, migration, apoptosis, qRT-PCR, and western blotting. The sequences of the shNC and shRheb are listed in supplemental (Supplemental Table 2).

### RNA extraction and Quantitative real-time PCR (qRT-PCR) analysis

Total RNA was extracted with Trizol Reagent (Sangon Biotech, Shanghai, China). Reverse transcription was performed with the RT-PCR Kit (Takara Biotech, Otsu, Japan) and PrimeScriptTM RT reagent Kit (Takara). qRT-PCR was carried out with SYBR Premix Ex Taq™ (Takara) according to the manufacturer's protocols. U6 snRNA and 18S rRNA were used as the endogenous controls for miRNA and mRNA. Results were expressed using the relative quantification (2^-ΔΔCt^) method. The primer sequences which related in this paper are shown in Supplemental Table 2.

### Cell proliferation assay and colony formation assay

Cell proliferation was determined with CCK-8 assay (Dojindo, Tokyo, Japan). Briefly, cells were plated in a 96-well microplate at a density of 2×10^3^ cells per well and incubated at 37˚C in a 5% CO_2_ humidified environment. Each sample was set with 4 or 5 replicates wells. After cultured for 24, 48, and 72 h, we added 8 μL CCK-8 solution and 92 μL serum-free medium to each well, and then detected after incubated for 2.5 h. Light absorbance at 450 nm was measured daily with an enzyme-linked analyzer, FLx8 (BioTek, Vermont, USA).

Cells were plated at 400 cells per 35-mm tissue culture dish and incubated for 2 weeks at 37˚C in a 5% CO_2_ humidified environment. Colonies were then fixed with methanol, stained with crystal violet (0.5% w/v), and counted. Each experiment with duplicates was performed independently at least thrice.

### Cell migration assay

Wound healing assay: A549 and H1299 were seeded at 2×10^5^ cells per well in 24-well plate and allowed to reach confluence. A single-scratch wound was introduced through the middle of each well with a sterile pipette tip. Cell migration across the margins was assessed and photographed by microscope after 24 h.

Transwell assay: Cell transwell assay was performed using 24-well transwells (6.5-mm pore size, Corning Life Sciences). Cells were transfected with 50 nm miRNA mimic. After 24 h, the cells were added to the upper chamber of each migration well in 100 μL FBS-free medium. 500 μL DMEM with 10% FBS was added to the lower part of the chamber and migration was allowed to occur for 24 h. The cells on the filter surface were fixed with methanol, stained with crystal violet, and photographed with a phase-contrast inverted microscope (Nikon, Tokyo, Japan). Experiments with triplicates were performed independently at least thrice.

### Cell apoptosis analysis

Cell apoptosis analysis was carried out as previously described 29. An Annexin V-fluorescein isothiocyanate (FITC) apoptosis detection kit (BD Pharmingen, San Diego, CA, USA) was used according to the manufacturer's instruction in order to determine the level of cell apoptosis. A549 and H1299 cells were resuspended in 1×binding buffer solution with Annexin V-FITC and propidium iodide (PI) , incubated at room temperature for 15 min in the dark. Apoptotic cells were analyzed using a MoFlo XDP flow cytometer (Beckman Coulter, Inc., Brea, CA, USA). Experiments with triplicates were performed independently at least thrice.

### Protein extraction and Western blot analysis

Total protein was extracted from the cells using RIPA lysis buffer (CWBIO, Beijing, China) and quantified with a Protein BCA Assay Kit (Bio-Rad, Hercules, California, USA). The protein was then separated by sodium dodecyl sulfatepolyacrylamide gel electrophoresis (SDS-PAGE) and transferred to a polyvinylidene difluoride (PVDF) membrane (Millipore Corporation, Billerica, MA, USA). The membrane was then blocked with 5% powdered milk at room temperature for 1 h, followed by incubation with rabbit anti-Rheb (13879S) and anti-GAPDH (14C10) antibodies (1:1000, Cell Signaling Technology, Danvers, MA, USA) overnight at 4˚C. After washing and incubating with a goat-anti-rabbit secondary antibody conjugated to horseradish peroxidase (HRP) (1:1000, Cell Signaling Technology), protein bands were detected with a chemiluminescent HRP substrate (Millipore Corporation) and analyzed by Image Lab analysis software (Bio-Rad).

### Dual-luciferase reporter gene assay

The common targets of miR-199a-3p/5p were screened based on TargetScan, miRWalk, PITA, and miRanda. 3′-UTR luciferase plasmids were constructed as follows: The 3′-UTR of Rheb containing the predicted binding site of miR-199a-3p and miR-199a-5p were respectively cloned into the pGL3 vector (Promega, Madison, USA) and named as Rheb-3′-UTR. Rheb-3′-mUTR plasmid containing the mutated binding site was also constructed. Site-directed mutagenesis was performed by quick-change-PCR with mutated primer pairs and Pfu polymerase (Takara, Dalian, China). Recombinant expression vectors were confirmed by sequencing (Sangon Biotech). Primer sequences are listed in Supplemental Table 2.

HEK293T cells were cultured in 24-well plate and transiently co-transfected with 200 ng of luciferase vector Rheb-3′-UTR or Rheb-3′-mUTR, and a final concentration of 100 nM of miR-199a-3p/5p mimic, or NC mimic, with 20 ng of plasmid expressing the renilla luciferase gene (pRL, Promega) as a control for transfection efficiency. Cells were lysed and luciferase activity was assayed with an Orion II Microplate Illuminometer (Titertek-Berthold, South San Francisco, USA) after 48 h post-transfection. Relative activities were expressed as the fold-change in luciferase activity following normalization to renilla luciferase activity.

### Immunohistochemistry Assay

Tumor growth was assessed by immunohistochemical (IHC) staining of Ki67 to quantitate the growth index, N-cadherin, and E-cadherin to quantitate the migration index. Target gene for miR-199a-3p/5p and Rheb was also measured by immunohistochemistry. We assessed tumor growth with immunohistochemical staining of tumor biopsies were fixed with formalin, embedded in paraffin, and cut into sections of about 4 μm. Samples were then deparaffinized and dehydrated with xylene and graded alcohols, and subsequently rehydrated with demineralized water. Immunohistochemistry was performed using microwave pre-treatment of slides for antigen retrieval. Primary antibodies against Ki67, Rheb, N-cadherin, and E-cadherin (1:500, Cell Signaling Technology) were applied together with goat anti-rabbit horseradish peroxidase (HRP)-conjugated antibodies proteins were visualized in situ with a 3, 3′-diaminobenzidine reaction solution.

### Lentivirus construction and infection

Lentivirus construction and infection performed as previously described. In brief, pri-miR-199a sequence was digested with BamH I and Xhol I, which then cloned into the pLenti vector (Invitrogen, Carlsbad, CA, USA) to form pLenti-miR-199a. pLenti-miR-199a or pLenti vector was co-transfected into HEK293T with psPAX2 and pMD2G. Viral particles were collected 48 h and 72 h later, centrifuged them together at 4000 rpm for 5 min at 4 degree, and filtered with 0.45 μm filter. A549 cells were then infected with the viral particles, transfected with pLenti-miR-199a, or pLenti, were sorted for green fluorescence via flow cytometry.

### Enzyme-linked immunosorbent assay (ELISA)

The protein levels of IL-6 and TNF-α in the conditional media were measured with the mouse IL-6 and TNF-α DuoSet ELISA kit (eBioscience) according to the manufacturer's instructions. A microplate reader (Synergy4; BioTek, Winooski, VT, USA) was used to read the absorbance at 450/570 nm.

### Tumor xenograft assay

Female nude mice were purchased from the Slaccas Laboratory Animal Center (Shanghai, China) and maintained under specific-pathogen-free conditions. To establish the subcutaneous tumor xenograft model, 6-8 weeks mice were randomly assigned into 2 groups (8 mice per group), and each nude mice was injected subcutaneously in the right flank with 5×10^6^ A549 cells (resuspended in 100 μL DMEM medium) pLenti-miR-199a. Tumor diameters were measured weekly with calipers, and tumor volumes were calculated using the following formula: volume = length× width^2^/2. Upon conclusion of the experiment, the mice were sacrificed and primary tumors were excised and weighed. To investigate the metastatic ability, mice were injected with 2.5×10^6^ cells (resuspended in 200 μL DMEM medium) via the tail vein, and seven weeks following the injection, the mice were sacrificed and lung tissues were isolated. Evaluation of lung tissue weights was used to quantify metastasis. Six tumor tissues were subjected to serial sectioning and IHC. Six lung tissues were subjected to serial sectioning and then haematoxylin and eosin (HE) staining. Pathological changes were observed under a light microscope.

All animal experiments were carried out in accordance to the Institutional Animal Care and Use Committee of Shanghai University (Shanghai, China). All efforts were made to minimize suffering.

### Establishment of Patient-derived xenograft (PDX) model

Fresh tumor tissues were obtained from Shanghai Chest Hospital and with the patient's informed consent. Fresh tumor tissue was placed in ice bath in sterile RPMI 1640 medium and transplanted subcutaneously (s.c.) into the left flank of anaesthetized 6-week-old nude mice within 2 h. Mice were maintained under sterile and controlled conditions (22°C, 50% relative humidity, 12 h light-dark cycle, autoclaved food and bedding, acidified drinking water). Tumor growth was measured in 2 dimensions with a caliper. Tumor volumes (TV) were determined by the formula: TV = length× width^2^/2. When tumor volumes reached 200 mm^3^, tumor tissues were passed on to the next generation until they reach the third generation. We used the third generation mice to validation of miR-199a for tumor suppression through agomiR-199a-3p/5p. Xenograft material was snap frozen and stored at -80°C or processed to formalin fixed, paraffin embedded (FFPE) blocks.

### Statistical analysis

Results were expressed as the mean ± standard error of the mean (SEM). Student's t-test between different groups of each experiment and one-way analysis of variance (ANOVA) for three or more group comparisons was used to determine the significance of differences. A *p*-value of < 0.05 was considered statistically significant. The relationship between Rheb and miR-199a-3p, miR-199a-5p was tested using Pearson's correlation and linear regression. Statistical analyses were performed with SPSS v.19.0 software and GraphPad Prism 8.0 software. *, *P* < 0.05; **,* P* < 0.01; ***, *P* < 0.001.

## Results

### MiR-199a-3p and miR-199a-5p are downregulated in NSCLC

**Figure 1:**
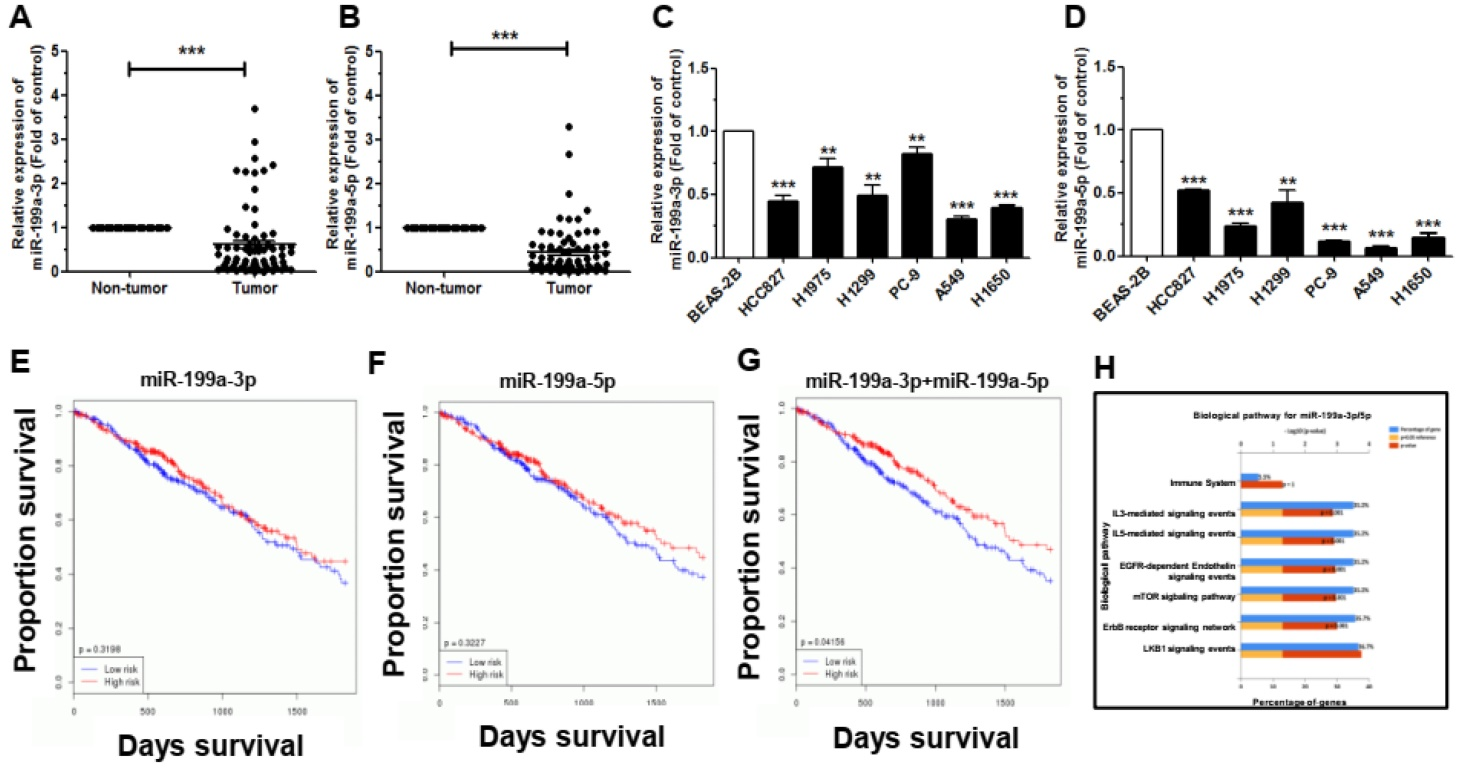
** MiR-199a-3p/5p is downregulated in NSCLC tissues and human cell lines, which associated with tumor survival. (A)** miR-199a-3p expression in NSCLC tissues detected by qRT-PCR. **(B)** miR-199a-5p expression in NSCLC tissues detected by qRT-PCR. **(C and D)** The expression levels of miR-199a-3p/5p in the six human NSCLC cell lines. BEAS-2B cells were used for the normal control comparison. **(E)** The effect of miR-199a-3p expression levels on the Kaplan-Meier curve for TCGA database in lung cancer patients. **(F)** The effect of miR-199a-5p expression levels on the Kaplan-Meier curve for TCGA database in lung cancer patients. **(G)** The combined effect of miR-199a-3p and miR-199a-5p expression levels on the Kaplan-Meier curve for TCGA database in lung cancer patients. **(H)** The biological pathway of miR-199a-3p/5p was analyzed using FunRich 3.1. *P<0.05, **P<0.01 and ***P<0.001.

To explore the expression of miR-199a-3p/5p in NSCLC tissues, we analyzed and compared in 74 pairs of lung cancer patient tissue samples and their corresponding para-cancerous tissues. This analysis revealed that the miR-199a-3p/5p expression level in NSCLC tissue samples was significantly lower than that in corresponding non-tumor tissues samples (Figure 1A, 1B). In these samples, the downregulation of miR-199a-3p was related to pathological stage, tumor size, and sex. The miR-199a-3p expression level in tissue samples from tumors in III/IV stage, > 3 cm in size and in female was higher than that in I/II stage, ≤ 3 cm in size and in male in tissue samples, separately (Supplementary Figure S1A-S1C). Yet, the expression of miR-199a-5p was not significantly related to pathological stage, tumor size, and sex (Supplementary Figure S1D-S1F). We also analyzed the expression level of miR-199a-3p/5p in NSCLC cell lines. Results showed that, compared with the normal bronchial epithelium cells (BEAS-2B), miR-199a-3p/5p was significantly downregulated in most of the NSCLC cell lines (Figure 1C, 1D). Another aspect, through TCGA database, we analysis miR-199a-3p/5p survival with patient samples, the results showed that miR-199a-3p/5p has a well prognosis (Figure 1E, 1F). What's more, survival rate is better under the combined effect of miR-199a-3p and miR-199a-5p (Figure 1G). Then, using FunRich 3.1 to analyze, the biological pathway of miR-199a-3p/5p might be associated with mTOR signaling pathway and inflammatory factors (Figure 1H). These results suggested that miR-199a-3p/5p may suppress NSCLC progression and directly predicted the patient overall survival rate.

### MiR-199a-3p and miR-199a-5p suppress the proliferation and migration of NSCLC, and promotes cell apoptosis

**Figure 2:**
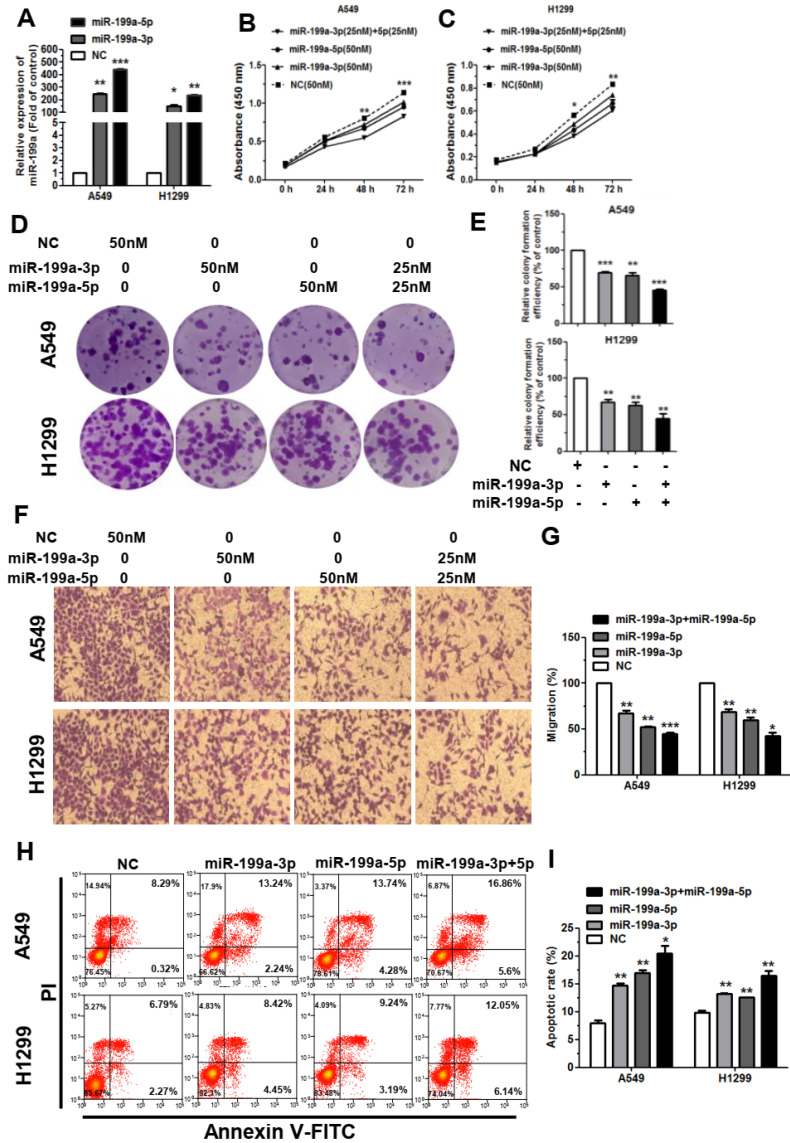
** MiR-199a-3p/5p could inhibit the proliferation and migration of NSCLC, and promote cell apoptosis. (A)** Upregulation of miR-199a-3p/5p following transfection with 50 nmol miR-199a-3p/5p mimic in A549 and H1299 cells for 48 h. **(B and C)** Cell proliferation ability of A549 and H1299 cells transiently transfected with miR-199a-3p/5p mimic measured by CCK-8 assay, testing in 24 h, 48 h, 72 h. **(D and E)** Colony formation assay in A549 and H1299 cells transfected with miR-199a-3p/5p mimic or NC. Representative images and quantitative data are shown. **(F and G)** Transwell migration assay performed after transfection of A549 and H1299 cells with miR-199a-3p/5p mimic or NC for 24 h. The migrated cells were stained with crystal violet and photographed. Migrated cells were counted and analyzed.** (H and I)** The rate of apoptosis was analyzed by flow cytometry following transfection with miR-199a-3p/5p mimic or NC in A549 and H1299 cells. Each assay was performed in triplicate. *P<0.05, **P<0.01 and ***P<0.001.

We first used the miR-199a-3p/5p mimic to increase the expression of miRNA in A549 and H1299 cells. Through qRT-PCR analysis, we found that transfection of miR-199a-3p/5p mimic upregulated the RNA level of miR-199a-3p/5p in A549 and H1299 cells compared with negative control (NC) mimic group (Figure 2A). Then, we investigated the cellular function of miR-199a-3p/5p in NSCLC. First, the cell proliferation was dramatically restrained after transfected with miR-199a-3p/5p mimic by cell counting kit 8 (CCK-8) and colony formation assays in A549 and H1299 cells (Figure 2B-2E). It's noteworthy that when miR-199a-3p and miR-199a-5p act together, they inhibit proliferation more obviously. Secondly, we examined the effect of miR-199a-3p/5p on the migration of NSCLC cells *in vitro* with wound healing and transwell assays. The results indicated that miR-199a-3p/5p could reduce the migration of A549 and H1299 cells in the transwell assay (Figure 2F, 2G), which migrated toward the wound at a much slower rate than the NC group cells in the wound healing assay (Supplementary Figure S2).

To further elucidate the function of miR-199a-3p/5p, we performed flow cytometry. Results showed that the apoptotic rate was promoted after transfected A549 and H1299 cells with miR-199a-3p/5p and NC mimic (Figure 2H, 2I). While transfected with miR-199a-3p/5p inhibitor can promote the proliferation and migration of the A549 and H1299 cell lines (Supplementary Figure S3A-S3G), the cell apoptosis was markedly attenuated (Supplementary Figure S3H, S3I). Together, these results indicated that miR-199a-3p/5p could significantly inhibit cell proliferation and migration, as well as promote apoptosis in A549 and H1299 cell lines.

### MiR-199a-3p and miR-199a-5p both directly target Rheb

**Figure 3:**
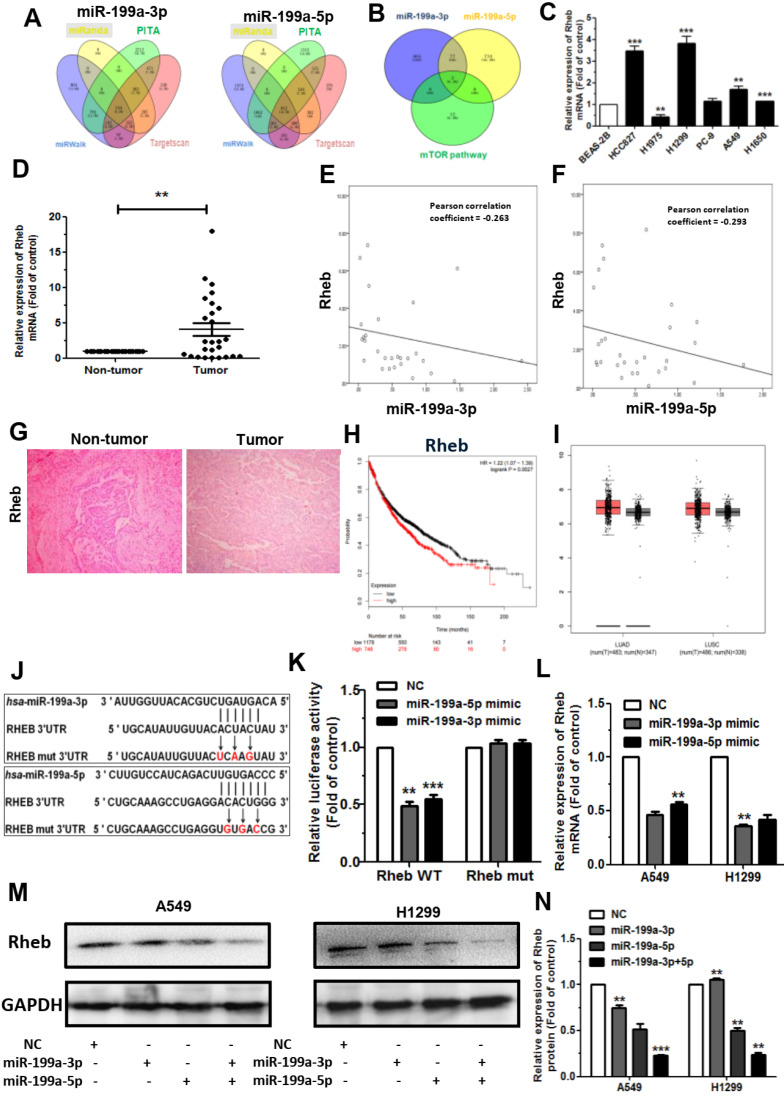
** Rheb is a common directly target of miR-199a-3p and miR-199a-5p. (A)** TargetScan, miRanda, PITA and miRWalk were used to predict direct target genes of miR-199a-3p and miR-199a-5p. **(B)** Combined with mToR signaling pathway gene, screening out the target gene of Rheb. **(C)** The expression levels of Rheb in human NSCLC cell lines. BEAS-2B cells were used for the normal control comparison.** (D)** The relative expression of Rheb mRNA from 74 primary NSCLC clinical tissue samples and their non-tumor tissues as measured by qRT-PCR.** (E and F)** There was a negative correlation between miR-199a-3p/5p and Rheb, according to the Pearson Correlation Coefficient.** (G)** Performed by immunohistochemistry, the relative expression of Rheb from NSCLC clinical tissue samples and its non-tumor tissues.** (H)** The effect of Rheb expression levels on the overall survival of 1926 lung cancer patients was analyzed. Kaplan-Meier plots were generated using a Kaplan-Meier Plotter. **(I)** Expression level of Rheb in LUAD and LUSC tissues through GEPIA analysising.** (J)** Rheb WT 3'-UTR contains predicted miR-199a-3p/5p binding site. The data shows alignment of miR-199a-3p/5p with Rheb WT 3'-UTR and arrows indicate mutagenesis nucleotides.** (K)** Dual luciferase assay in HEK293T cells co-transfected with WT or mut Rheb 3'-UTR luciferase vectors and miR-199a-3p/5p mimic or NC mimic.** (L)** The mRNA levels of Rheb were detected by qRT-PCR in A549 and H1299 cells transfected with miR-199a-3p/5p mimic or NC after 48 h. **(M and N)** Protein levels of Rheb in miR-199a-3p/5p-overexpressing cells. Representative images (M) and quantitative data (N) are shown. *P<0.05, **P<0.01 and ***P<0.001.

To explore the molecular mechanism by which miR-199a-3p/5p suppresses NSCLC progression, we predicted the potential target genes using TargetScan (http://www.targetscan.org/vert_72/), miRanda (http://miranda.org.uk/), PITA (https://genie.weizmann.ac.il/pubs/mir07/mir07_data.htmL), and miRWalk (http://zmf.umm.uni-heidelberg.de/apps/zmf/mirwalk2/) (Figure 3A). Based on FunRich 3.1 analysis, Rheb was identified as both directly target of miR-199a-3p and miR-199a-5p, which involved in mToR signaling pathway (Figure 3B).

Then, we detected the expression of Rheb in human lung cancer cell lines by qRT-PCR, which verified that Rheb was upregulated in most cell types (Figure 3C). Further analysis showed that Rheb was also upregulated in 74 primary NSCLC clinical tissue samples compared with their non-tumor tissues (Figure 3D). Through statistical analysis using the Pearson Correlation Coefficient, there was a negative correlation between the relative Rheb expression and miR-199a-3p/5p in the NSCLC tissues samples (Figure 3E, 3F). Similarly, GEPIA (http://gepia.cancer-pku.cn/index.html) also found a negative correlation (Supplementary Figure S4A, S4B). Hence, it is speculated that miR-199a-3p/5p inhibits the development of NSCLC by suppressing Rheb. Additionally, we found that the expression level of Rheb was upregulated in the carcinoma compared to the paracancer by performing immunohistochemistry (Figure 3G). The Kaplan-Meier Plotter online database showed that high expression of Rheb had a lower overall survival rate among the 1926 NSCLC cases (Figure 3H). Subsequently, GEPIA analysis showed that in lung adenocarcinoma (LUAD) and lung squamous cell carcinoma (LUSC) tissues, Rheb was also highly expressed in cancer patients compared with normal tissues (Figure 3I). These cases further showed that Rheb expression levels increased with increasing tumor stage in LUAD and LUSC tissue samples (Supplementary Figure S4C, S4D).

To confirm investigate whether or not Rheb is a both directly target of miR-199a-3p and miR-199a-5p, we performed the luciferase reporter assay. The wild-type and mutant binding sites of miR-199a-3p and miR-199a-5p to Rheb are shown in Figure 3J. HEK293T cells were co-transfected with pGL3- Rheb wild-type or mutant 3'-UTR along with miR-199a-3p/5p mimic and pRL (plasmid expressing a renilla luciferase gene) vector. Results showed that the relative luciferase activity was remarkably decreased in Rheb wild-type 3'-UTR-transfected cells compared to Rheb mutant 3'-UTR-transfected cells, suggesting a potential inhibitor binding activity of miR-199a-3p/5p to Rheb 3'-UTR. Inversely, cotransfection of miR-199a-3p/5p with Rheb 3'-mUTR (pGL3-Rheb mut 3'-UTR) resulted in no significant change in luciferase activity (Figure 3K). The above results were further confirmed by using qRT-PCR and western blotting (WB) to determine the mRNA and protein levels of Rheb, showing that miR-199a-3p/5p decreased the mRNA and protein level of Rheb in A549 and H1299 cells (Figure 3L, 3M, 3N).

### Downregulation of Rheb reduces the proliferation and promotes apoptosis in NSCLC

**Figure 4:**
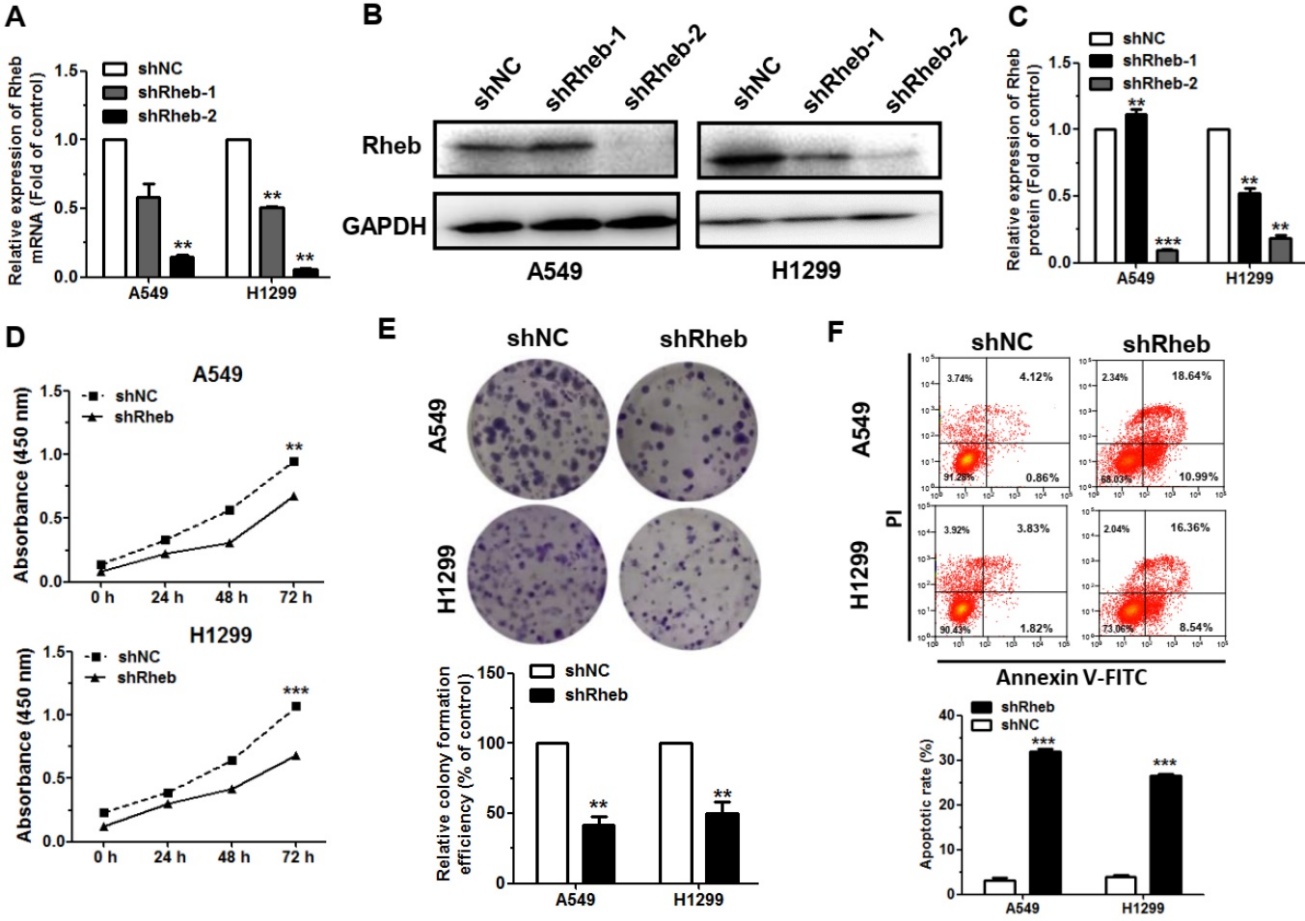
** Knockdown of Rheb reduces cell proliferation and promotes apoptosis in NSCLC. (A-C)** The levels of Rheb mRNA and protein in A549 and H1299 cells transfected with Rheb shRNA (shRheb-1, shRheb-2) were measured by qRT-PCR and western blot in 48 h, respectively. **(D and E)** Proliferation of A549 and H1299 cells transfected with shRheb-2 was determined by CCK-8, testing in 24 h, 48 h, 72 h (D) and colony formation (E).** (F)** The rate of apoptosis of A549 and H1299 cells transfected with shRheb-2 was analyzed by flow cytometry. **P<0.01 and ***P<0.001.

To validate the regulatory role of Rheb in NSCLC, Rheb was silenced by using short hairpin RNA (shRNA; shRheb-1, shRheb-2). Our results revealed that the expression levels of Rheb mRNA and protein were significantly downregulated in NSCLC cells, which transfected with shRheb-1 and shRheb-2 compared with the shNC (Figure 4A, 4B, 4C). Due to the high downregulation efficiency of shRheb-2, subsequent validation experiments were conducted using shRheb-2. Results revealed that the proliferation of cells transfected with shRheb was significantly decreased, compared with that of the shNC transfected cells (Figure 4D, 4E). Furthermore, flow cytometric analysis of cell apoptosis showed that transfect with shRheb has a significant increase in the rate of apoptosis in A549 and H1299 cells, compared with that of the shNC transfected cells (Figure 4F).

Rheb is known to be an activator of the mTOR signaling pathway and abnormal expression in many cancers clinically 30.Through ENCORI (http://starbase.sysu.edu.cn/index.php) database analysis, Rheb showed a positive correlation with mTOR (Supplementary Figure S5A). Then, we next verified that overexpression of miR-199a-3p and miR-199a-5p revealed little change in protein expression level of mTOR. However, mTOR phosphorylation was decreased in A549 and H1299 cells (Supplementary Figure S5B). In combination with the previous findings by functional analysis, miR-199a-3p/5p was found to be involved in regulating the mTOR signaling pathway.

Overall, these results demonstrated that Rheb knockdown and miR-199a-3p/5p upregulation exhibited similar functions in NSCLC cells. MiR-199a-3p/5p is involved in regulating the mTOR signaling pathway through Rheb. More information indicates that the tumor suppressive function of miR-199a-3p/5p is mediated partly by downregulation of Rheb.

### MiR-199a suppresses tumor growth in A549 xenograft and metastatic tumors

**Figure 5:**
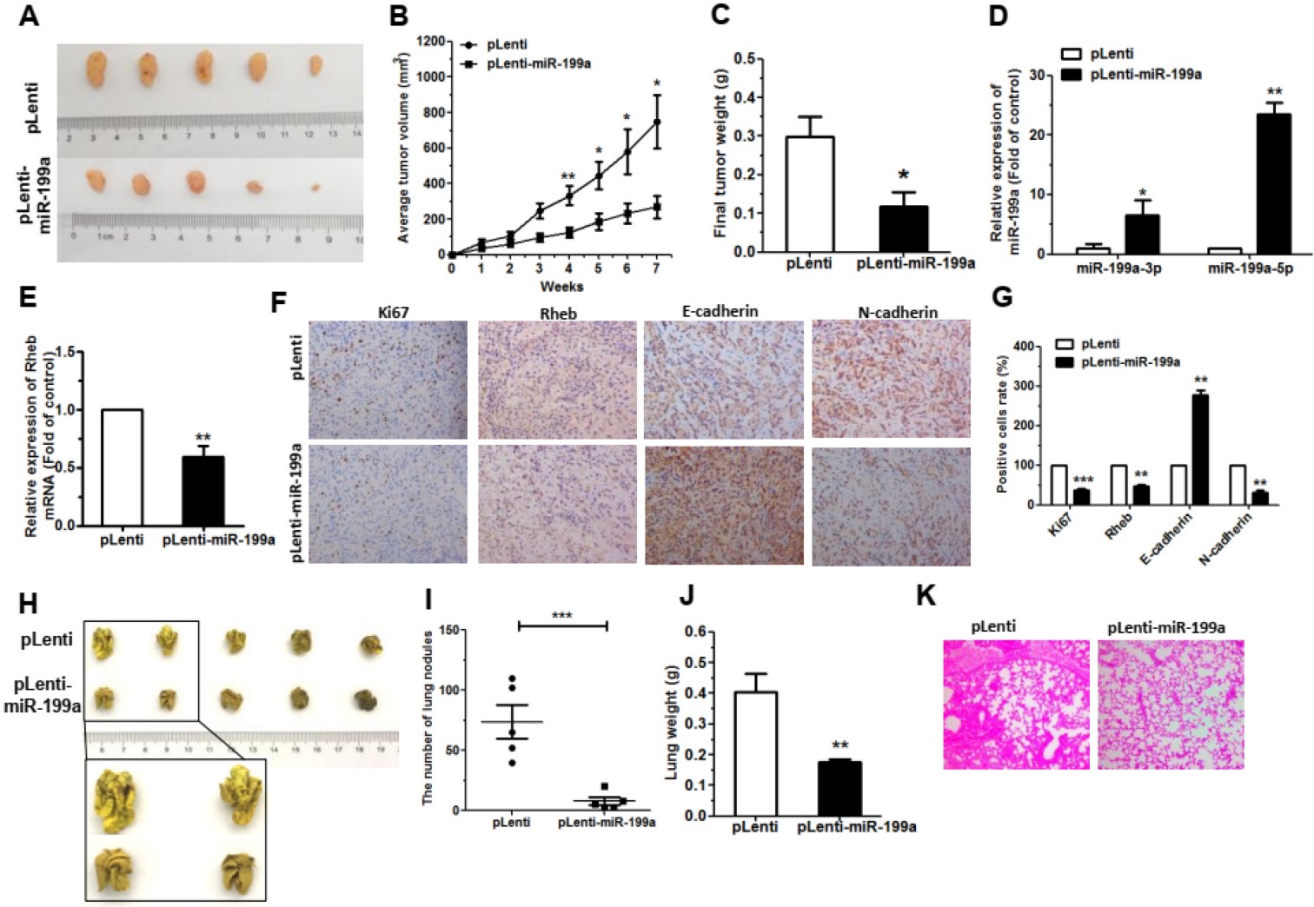
** miR-199a inhibited tumor growth and metastases *in vivo*. (A**) pLenti-miR-199a A549 cells and control cells (pLenti) were injected into nude mice by subcutaneous or tail vein injection. Images tumors isolated from mice after 7 weeks. **(B)** Tumor growth curves with volume measured every week after injection. **(C)** The weights tumors isolated from mice after 7 weeks. **(D)** The expression of miR-199a-3p/5p was detected by qRT-PCR in the mouse tumor tissues. **(E)** The expression of Rheb was detected by qRT-PCR in the mouse tumor tissues. **(F and G)** The expression of Ki67, Rheb, E-cadherin and N-cadherin in tumor tissues was measured by immunohistochemistry.** (H)** The lungs of mice with metastasis nodes are displayed. **(I and J)** The numbers of lungs nodes and weight of lungs of mice induced by pLenti-miR-199a A549 cells and pLenti cells are displayed. **(K)** Histopathology of metastases with HE staining. *P<0.05, **P<0.01 and ***P<0.001.

To investigate the tumorigenic role of miR-199a, we generated xenograft mouse models by subcutaneous injection of A549 cells with miR-199a-stably-overexpressing (pLenti-miR-199a) A549 cells (5 × 10^6^) or the control (pLenti), and subsequent tumors were assessed every week. The results showed that overexpression of miR-199a significantly suppressed tumor growth in mice (Figure 5A). The pLenti-miR-199a cells also exhibited obviously reduction in both tumor size and weight at 7 weeks post-implantation (Figure 5B, 5C). Moreover, the expression of miR-199a-3p and miR-199a-5p were upregulated in tumor tissues from the pLenti-miR-199a group when compared to the controls (Figure 5D). Conversely, a significant downregulation of Rheb expresses in tumor tissues from pLenti-miR-199a group (Figure 5E). This indicates that there is a negative correlation between miR-199a-3p/5p and Rheb. Next, using immunohistochemistry, we measured the expression of Ki67, Rheb, E-cadherin and N-cadherin in the xenograft tumor tissues. Results showed a significant downregulation in Ki67 and Rheb in the tumor tissues over-expression pLenti-miR-199a, accompanied by an induction of E-cadherin expression, but a decrease in the expression of N-cadherin (Figure 5F, 5G). These data indicated that miR-199a might inhibit tumor growth via regulating of Rheb *in vivo*, complementing the results of our functional* in vitro* studies.

To ascertain the function of miR-199a in tumor metastasis *in vivo*, A549 cells (2.5 × 10^6^) pLenti-miR-199a, or pLenti, were injected into the tail vein of nude mice. Seven weeks later, the number of the nodes eventually formed in the lungs of pLenti-miR-199a was significantly lower compared to the control group (Figure 5H, 5I). Statistical analysis showed that the weights of the lungs from mice with metastatic nodes in the pLenti-miR-199a cell group had shrunk about 0.53-fold compared with those in the control cell group (Figure 5J). By H&E staining under light microscopy used to observe pathological changes in the bilateral lungs. Similar to a normal lung tissue nests, the pLenti-miR-199a group formed a larger and sparser reticular structure whereas the tumor nests derived from control cells exhibited an area of lung tissue destruction and/or necrosis (Figure 5K).

### MiR-199a-3p and miR-199a-5p can restraint the ratio of tumor growth in A549 xenograft through PDX model

**Figure 6:**
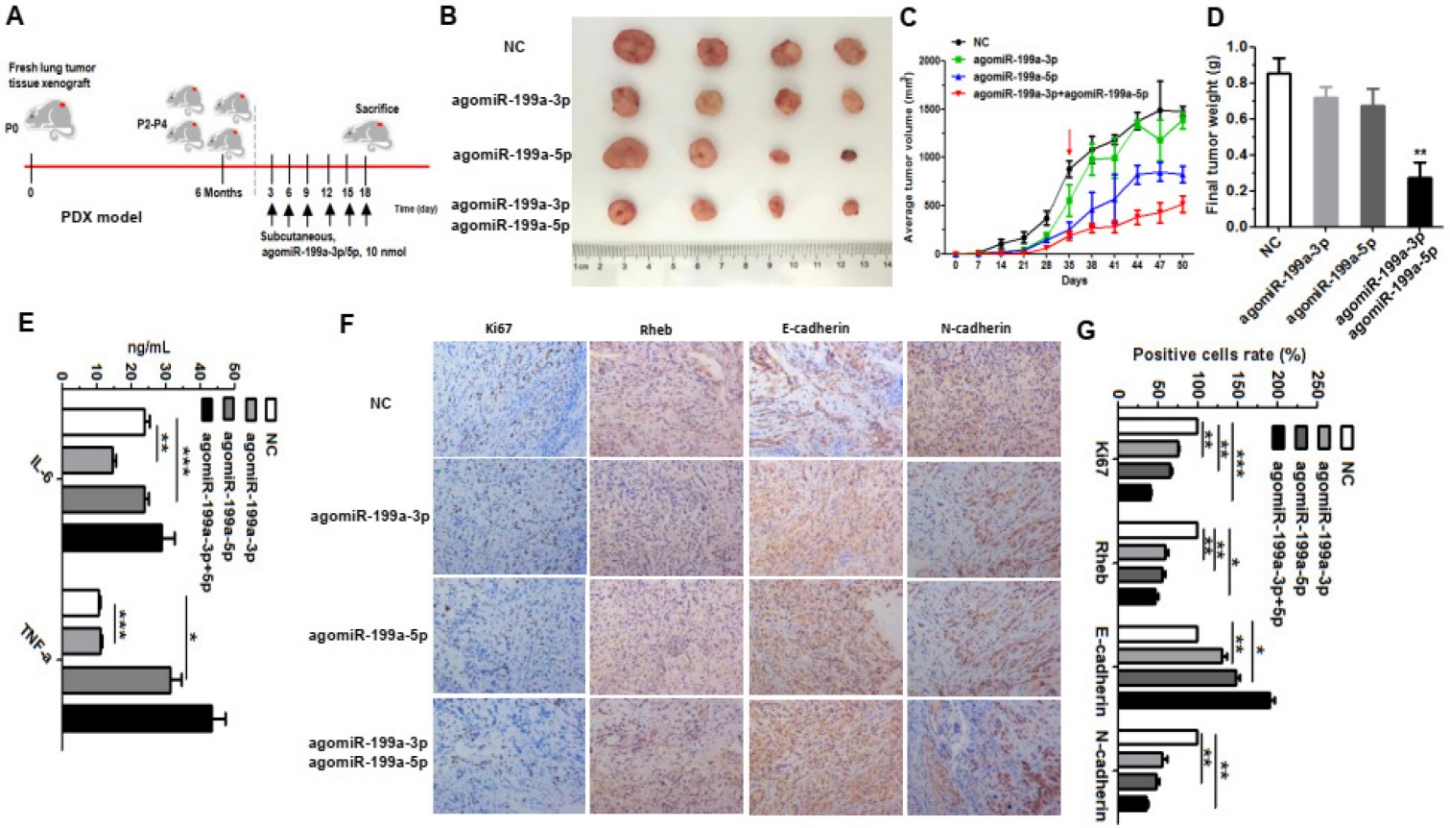
** miR-199a suppressed tumor growth in PDX model. (A)** Using fresh tumor tissue to establish PDX model and treatment of agomiR-199a-3p/5p in the PDX model (8 mice per group).** (B)** Tumor images are displayed.** (C)** Tumor growth curves with volume measured every week with the 4th generation PDX nude mice set up. AgomiR-199a-3p/5p started treatment through subcutaneous injections when the 35th day and measured volume measured every 3 days. **(D)** The weights tumors isolated from mice. **(E)** The expression of IL-6 and TNF-α were detected by ELISA.** (F and G)** Using immunohistochemistry to measure the expression of Ki67, Rheb, E-cadherin and N-cadherin in tumor tissues. *P<0.05, **P<0.01 and ***P<0.001.

Patient-derived xenograft (PDX) tumor models provide a valuable platform for identifying new biomarkers and novel targets, assessing treatment response, and resistance mechanisms 31. To further explore the effects of miR-199a-3p and miR-199a-5p *in vivo,* we verified this with the PDX model using human fresh lung tumor tissue (Figure 6A). When the 4th generation PDX nude mice set up, we start subcutaneous injections. We first randomly divided nude mice into four groups, NC group, agomiR-199a-3p group, agomiR-199a-5p group and agomiR-199a-3p+ agomiR-199a-5p group. When the subcutaneous tumor reached proper size about 35 days, the tumor-bearing mice were treated with agomiR-199a-3p/agomiR-199a-5p around the formed subcutaneous tumor in different groups, being injected and measured tumor sizes every three days. Results showed that the groups injected agomiR-199a-3p and agomiR-199a-5p inhibited the ratio of tumors growth, this phenomenon was more pronounced with agomiR-199a-5p and agomiR-199a-3p acting together (Figure 6B, 6C, 6D). Moreover, we examined the expression of inflammatory factors in the serum of mice. Outcomes showed that interleukin- 6 (IL-6) expression was significantly upregulated in agomiR-199a-5p and agomiR-199a-3p+agomiR-199a-5p groups compared with NC group. The expression of IFN-α was also upregulated in agomiR-199a-3p, agomiR-199a-5p, and agomiR-199a-3p+agomiR-199a-5p groups compared with NC group (Figure 6E). This implies that miR-199a-3p/5p may be involved in the regulation of tumor immunity. In addition, we also detected the expression of Ki67, Rheb, E-cadherin, and N-cadherin in the tumor tissues through immunohistochemistry. Results showed that a significant downregulation in Ki67 and Rheb in the tumor tissues that injected agomiR-199a-3p and agomiR-199a-5p, accompanied by an induction of E-cadherin expression, but a deduction in the expression of N-cadherin (Figure 6F, 6G). These results further demonstrate that miR-199a-199a-3p/5p inhibits the growth of tumors.

### MiR-199a-3p and miR-199a-5p can increase the sensitivity of gefitinib in NSCLC

**Figure 7:**
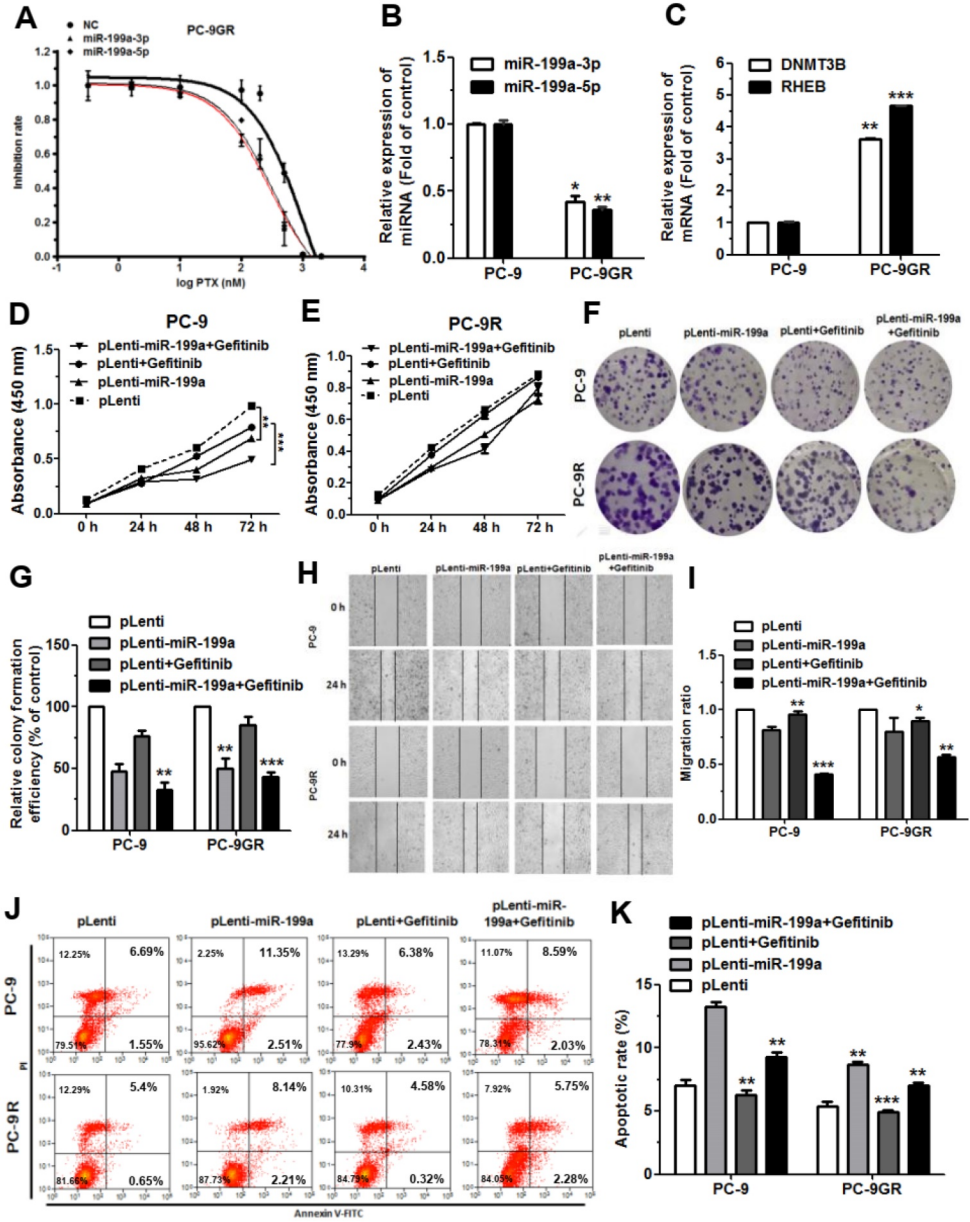
**miR-199a-3p/5p promoted the sensitivity of gefitinib in NSCLC. (A)** Half-inhibitory concentrations of gefitinib on miR199a-3p/5p highly expressed versus NC in PC-9GR cells, through set 10 concentration gradients. **(B)** The relative expression levels of miR-199a-3p/5p detected by qRT-PCR in PC-9 and PC-9GR cells.** (C)** The expression mRNA levels of DNMT3B and Rheb detected by qRT-PCR in PC-9 and PC-9GR cells. **(D-G)** The cell proliferation of PC-9 and PC-9GR cells with miR-199a-stably-overexpressing treatment with gefitinib, testing in 24 h, 48h, 72 h.** (H and I)** PC-9 and PC-9GR cells with miR-199a-stably-overexpressing treatment gefitinib were subjected to wound healing assay and images were taken at 0 h and 24 h. **(J and K)** The rate of apoptosis was analyzed by flow cytometry following treatment with gefitinib in PC-9 and PC-9GR cells with miR-199a-stably-overexpressing.*P<0.05, **P<0.01 and ***P<0.001.

In the course of tumor treatment, the development of resistance to many targeted drug or chemotherapeutic agents greatly hinders the effectiveness of tumor treatment. Based on the previous experiments, we next explored the effect of miR199a-3p/5p on gefitinib resistance in NSCLC cells. We used EGFR-T790M resistant mutant PC-9 Gefitinib Resistance (PC-9GR) cells to inquiring the half maximal inhibitory concentration (IC_50_) of gefitinib on miR199a-3p/5p high expression. The outcome shows that the IC_50_ of miR199a-3p and miR-199a-5p high expression groups were 290.6 nM and 331.4 nM, respectively, which are significantly lower than the control group (IC_50_: 1112 nM) (Figure 7A). This implies that high expression of miR-199a-3p/5p significantly improved its sensitivity to gefitinib. We also detected lower expression levels of miR199a-3p and miR199a-5p in PC-9GR than in PC-9 (Figure 7B). The methylation transferase DNMT3B showed a remarkable trend of high expression, leading to an elevated methylation level in the upstream promoter region of the miR-199a-3p/5p gene with oncogenic effects (Figure 7C). This may cause potentially epigenetic silencing, which we will continue to explore in future studies. But the expression level of Rheb was upregulated, which can activate the downstream mTOR signaling pathway (Figure 7C).

Subsequently, we constructed PC-9 and PC-9GR cells with miR-199a-stably-overexpressing (pLenti-miR-199a) by lentiviral methods. Gefitinib (50 nM) was administered to test the functional effects on PC-9 and PC-9GR in stable pLenti-miR-199a cell lines. Results indicated that after treatment gefitinib of the cells, PC-9 cells with high stable expression of miR-199a were subjected to greater inhibition of proliferation than PC-9GR compared to the control group (Figure 7D-7G). After the same administration, overexpression of miR-199a, PC-9 cells were subjected to a more pronounced degree of migration inhibition than that of PC-9GR (Figure 7H, 7I). Then, both PC-9 and PC-9GR cells with high miR-199a expression promoted apoptosis, but the apoptosis rate was more pronounced in the PC-9 group after treatment gefitinib (Figure 7J, 7K). Taken together, these results suggest that overexpression of miR-199a enhances the drug sensitivity of gefitinib in NSCLC.

## Discussion

**Figure 8:**
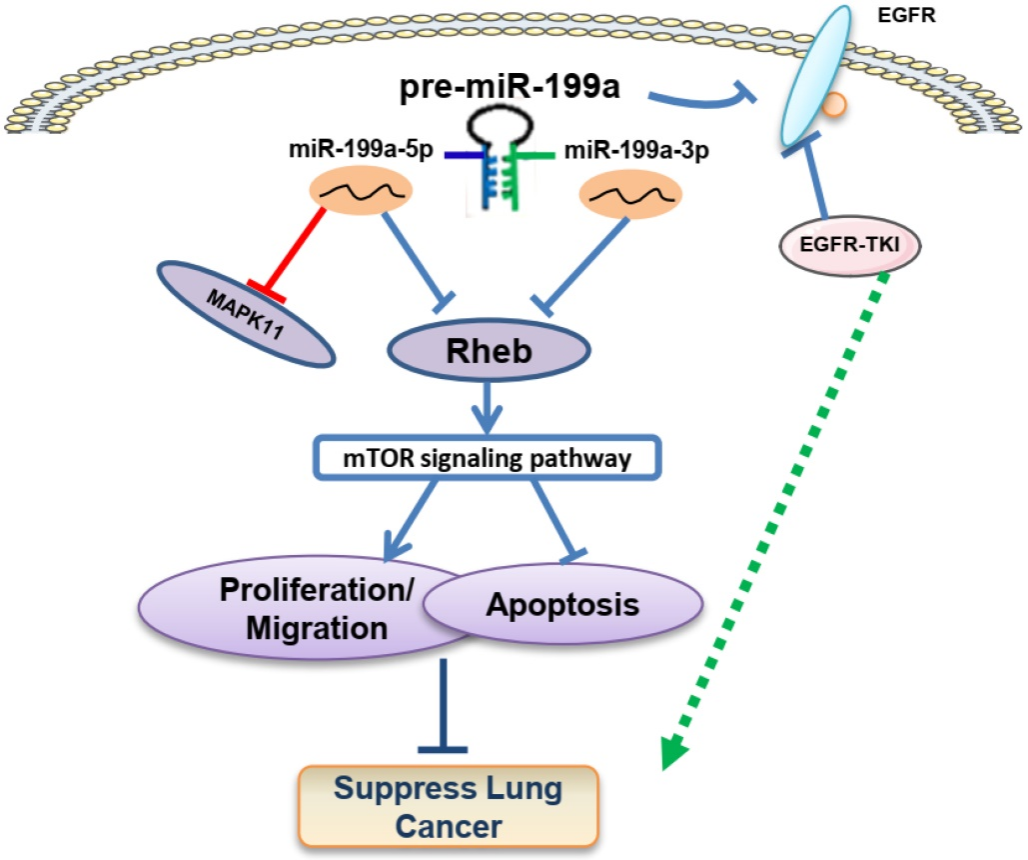
Possible mechanism of miR-199a/Rheb/mTOR Axis in NSCLC carcinogenesis. We present that miR-199a-3p/5p has the role of cancer suppressor via targeting Rheb/mTOR pathway and promotes the sensitivity of gefitinib in NSCLC.

MiRNAs, a class of small single-stranded RNA molecules that are highly conserved and unafraid of coding ability, can specifically degrade or inhibit the translation of target messenger RNAs and regulate the expression of target genes in the development of many tumors. Studies have found that miR-20a-5p accelerates the function of proliferation and invasion in NSCLC by targeting and downregulating KLF9 32. MiR-411 can promote NSCLC progression by targeting SPRY4 and TXNIP, and also promote tumor metastasis by inducing EMT (Epithelial-mesenchymal transition) 33. MiR-34a inhibits proliferation, migration, apoptosis, and cycle progression of lung cancer via targeting EGFR 29. In our previous research, we found that miR-199a-5p can suppress NSCLC through targeting MAP3K11 34. This study demonstrated that miR-199a-3p and miR-199a-5p showed a trend of low expression in tissue as well as cell lines in NSCLC. According to the bioinformatics analysis, miR-199a-3p and miR-199a-5p expression levels were strongly correlated with lung cancer stage progression and had an impact on the overall survival rate. MiR-199a-3p/5p overexpression inhibited cell proliferation, migration, and promoted apoptosis in NSCLC. Screening analysis in the database identified Rheb, a common target gene of miR-199a-3p and miR-199a-5p, involved in regulating the downstream mTOR signaling pathway. Experiments on mice showed that miR-199a inhibited tumor growth and metastasis, and promoted the expression of inflammatory factors IL-6 and IFN-α. Additionally, miR-199a-3p and miR-199a-5p enhanced the sensitivity of EGFR-T790M-induced gefitinib drugs in NSCLC. Overall, our findings suggest that the miR-199a/Rheb/mTOR axis may function as a tumor suppressor within this regulation network (Figure 8).

Up to now, miR-199a-3p and miR-199a-5p are differentially expressed in variety tumors and involved in biological processes such as tumor growth 35, metabolism 36, and metastasis invasion 37. They involved in the regulation of multiple signaling pathways, which acted an essential role in tumor prevention, early diagnosis, treatment and prognosis. Study has found that miR-199a-5p was associated with a poor prognostic phenotype and inhibited proliferation and metabolism of colorectal cancer by targeting ROCK1 38. *Ghosh* et al. discovered that miR-199a-3p inhibited tumor growth, migration, invasion and angiogenesis in hepatocellular carcinoma through targeting VEGFA, VEGFR1, VEGFR2, HGF and MMP2 39. MiR-199a-3p has a targeting relationship with AXL and negatively regulates the progression of osteosarcoma through AKT signaling pathway 40. MiR-199a-3p inhibits breast cancer cell migration and invasion through downregulation of the PAK4/MEK/ERK signaling pathway 41. In summary, miR-199a-3p and miR-199a-5p are involved in regulating the expression profile of tumor-related genes and they act as critical switches in gene expression.

Currently, early diagnosis and treatment of tumors have long been a hot topic in medicine and life sciences 42-44. *Zeng* et al. reported that upregulation miR‑199a‑5p inhibits autophagy and renders resistance to chemotherapeutic drugs in lung cancer cells by activating the PI3K/Akt/mTOR pathway and targeting p62 45. We will also continue to monitor the impact of the miR-199a/Rheb/mTOR axis on the development of resistance to the treatment of tumors in lung cancer, especially about EGFR-TKIs resistance. 50% of acquired resistance to EGFR-TKI is due to the presence of T790M mutation, which increases the affinity of the ATP-binding site for ATP and inhibits the binding of TKI to this site, resulting in drug resistance in patients 14, 46. More than 100 proteins have been found to interact with EGFR and specifically promote the activation of signaling pathways such as NF-κB, STAT, SRC, mTOR and MAPK 47.These signaling pathways are the "main switch" for the expression of inflammatory factors, such as TNF-α, IL-2, IL-6, monocyte chemotactic protein 1 (MCP-1), etc 48, 49. In another context, the inhibitors of mTOR signaling pathway mainly include rapamycin, its analogs sirolimus and everolimus. But the monotherapy effect of mTOR inhibitors in clinical responses is not significant due to feedback responses and crosstalk with other signaling pathway 50. Nevertheless, studies have shown that the combination of mTOR inhibitors with EGFR-TKIs, such as Erlotinib, has been effective in clinical trials 51. Combined with the inter-regulatory role of non-coding RNAs and signaling pathways, it is believed to be relevant for exploring the mechanism of cancer progression and precision medicine represented by targeted therapy.

In summary, our study demonstrated that miR-199a-3p and miR-199a-5p could inhibit the progression of NSCLC, providing a high potential for the complex regulatory network of miRNA-NSCLC to become a diagnostic marker and therapeutic agent for cancer.

## Supplementary Material

Supplementary figures and tables.

## Abbreviations

miRNA: microRNA
NSCLC: non-small cell lung cancer
mTOR: Mammalian target of rapamycin
Rheb: Ras homolog enriched in brain
EGFR: Epidermal growth factor receptor
SDS-PAGE: Sulfatepolyacrylamide gel electrophoresis
PVDF: Polyvinylidene difluoride
HRP: Horseradish peroxidase
CCK-8: Cell counting kit 8
ELISA: Enzyme-linked immunosorbent assay
PDX: Patient-derived xenograft
PC-9GR: PC-9 Gefitinib Resistance
EMT: Epithelial-mesenchymal transition
