# miR-199a-3p/5p regulate tumorgenesis via targeting Rheb in non-small cell lung cancer: Erratum

**DOI:** 10.7150/ijbs.86344

**Published:** 2023-07-27

**Authors:** Xiaomin Liu, Xianyi Wang, Binshu Chai, Zong Wu, Zhitao Gu, Heng Zou, Hui Zhang, Yanli Li, Qiangling Sun, Wentao Fang, Zhongliang Ma

**Affiliations:** 1 Lab for Noncoding RNA & Cancer, School of Life Sciences, Shanghai University, Shanghai 200444, China.; 2 Department of Thoracic Surgery, Thoracic Cancer Institute, Shanghai Chest Hospital, Jiaotong University Medical School,Shanghai 200030, China.

In our paper, the author noticed an error in Figure 6F. There is a non-subjective error in the image of IHC in “agomiR-199a-3p group” (Figure 6F). We checked the original data again and made sure that the conclusion of the article was not affected by the error. All authors have agreed to the erratum, and we apologize for any inconvenience caused by the negligence in our work.

Figure 6F should be corrected as follows.

## Figures and Tables

**Figure 6:**
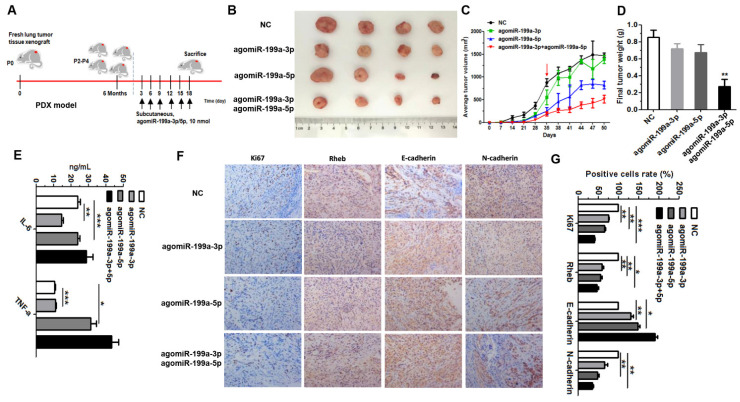
Correct image.

