# Supplementary material for: miR-199a-3p/5p regulate tumorgenesis via targeting Rheb in non-small cell lung cancer
Source: Int J Biol Sci. 2022 Jun 27;18(10):4187–202. doi: 10.7150/ijbs.70312 (PMC9274486; doi:10.7150/ijbs.70312)
Supplement: Supplementary file 1 — Supplementary figures and tables. [file ijbsv18p4187s1.pdf]

Figure Supplementary

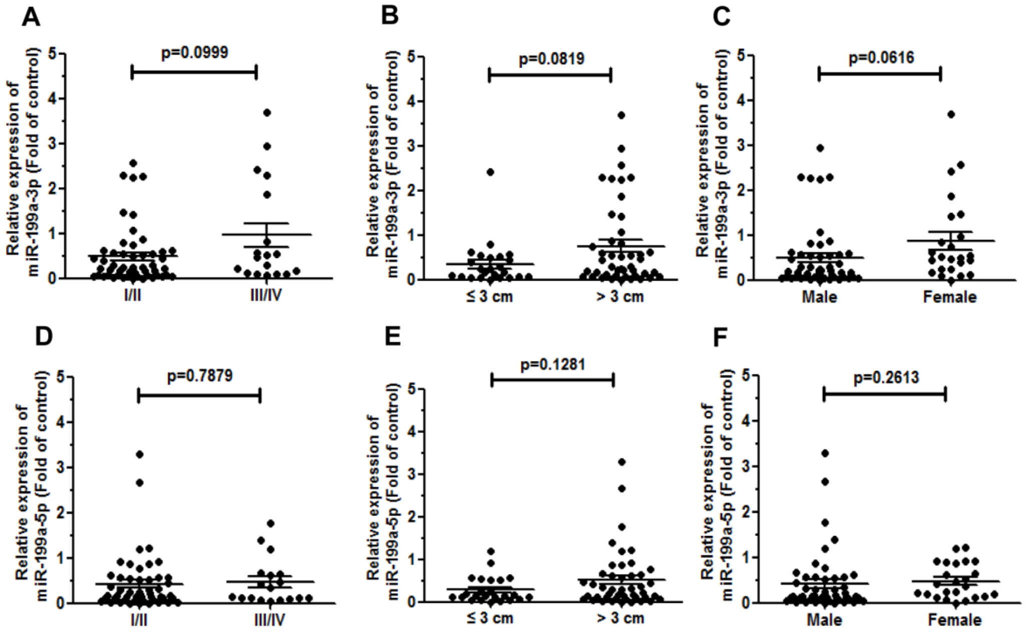

Figure Supplementary 1. (A-C) Expression of miR-199a-3p as related to tumor size, sex and pathological stage. **(D-F)** Expression of miR-199a-5p as related to tumor size, sex and pathological stage.

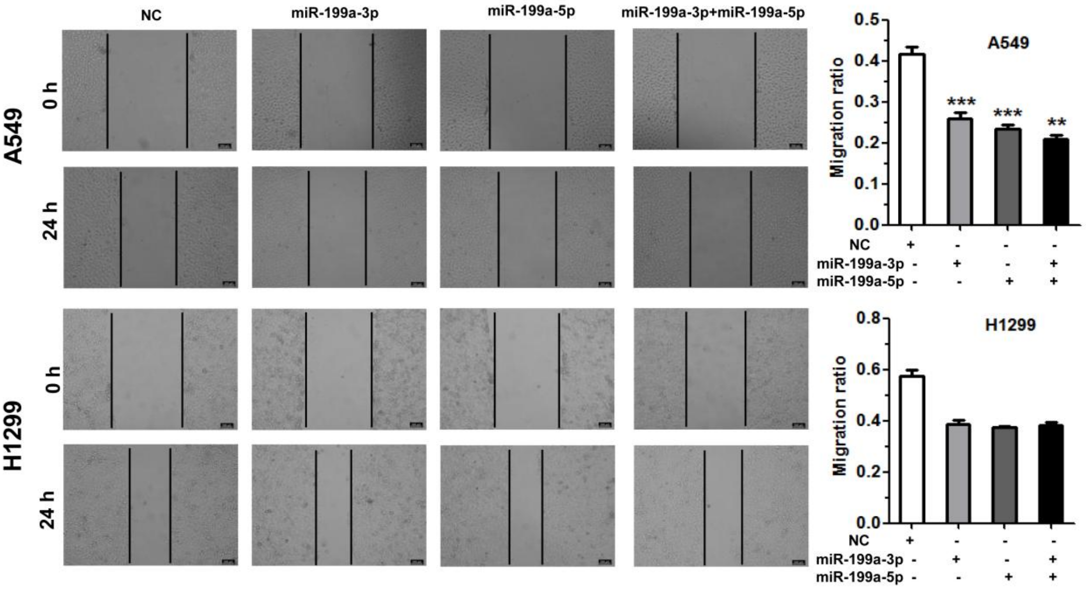

Figure Supplementary 2. A549 and H1299 cells transfected with miR-199a-3p/5p mimic or NC were subjected to wound healing assay and images were taken at 0 h and 24 h.

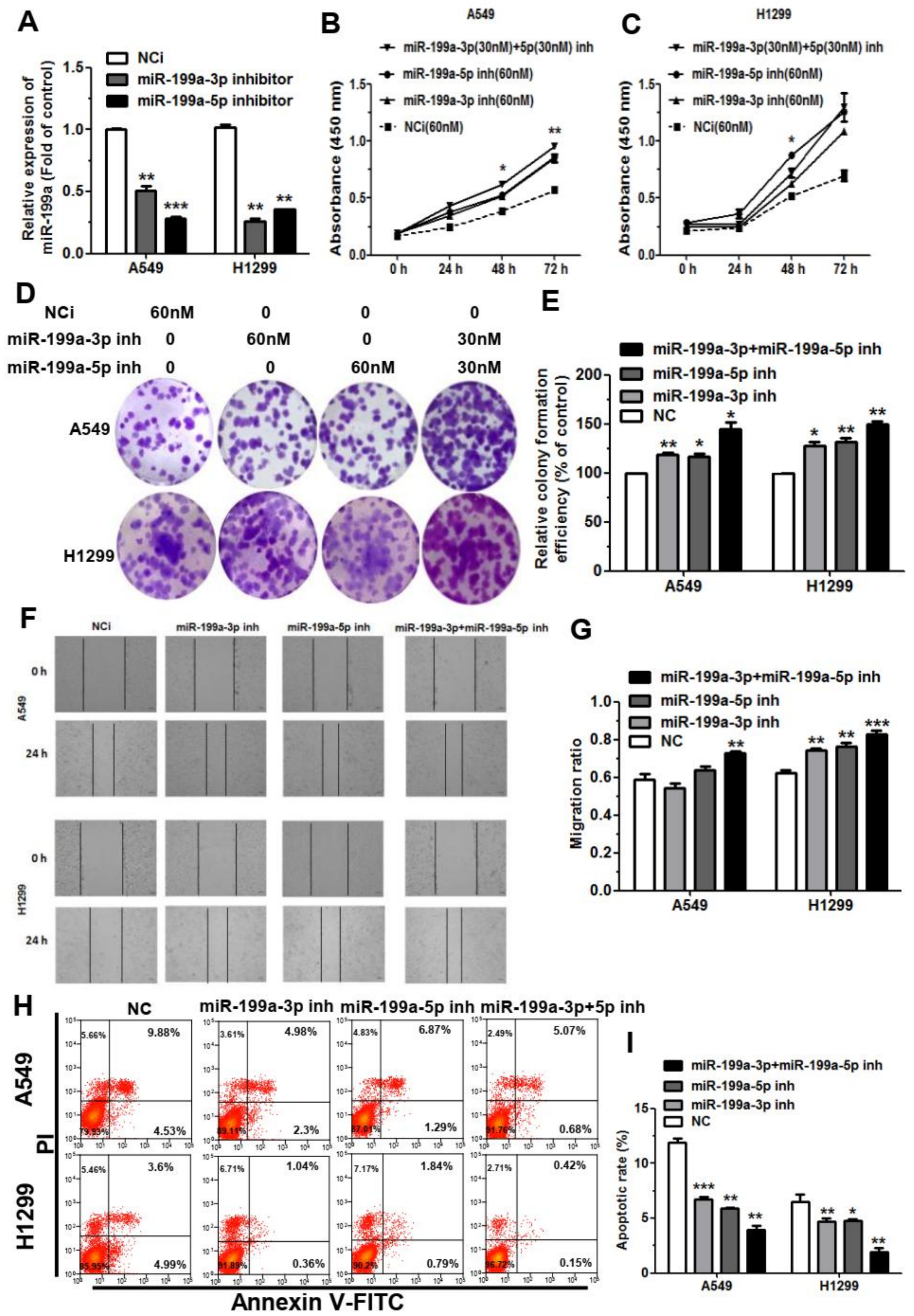

Figure Supplementary 3. Downregulation of miR-199a-3p/5p could promote the proliferation and migration of NSCLC, and suppress cell apoptosis. (**A**) Downregulation of miR-199a-3p/5p following transfection with 60 nmol miR-199a-3p/5p inhibitor in A549 and H1299 cells. (**B and C**) Cell proliferation ability of A549 and H1299 cells transiently transfected with miR-199a-3p/5p inhibitor measured by CCK-8 assay. (**D**

and E) Colony formation assay in A549 and H1299 cells transfected with miR-199a-3p/5p inhibitor or NC. Representative images and quantitative data are shown. (**F and G**) A549 and H1299 cells transfected with miR-199a-3p/5p inhibitor or NC were subjected to wound healing assay and images were taken at 0 h and 24 h. (**H and I**) The rate of apoptosis was analyzed by flow cytometry following transfection with miR-199a-3p/5p inhibitor or NC in A549 and H1299 cells. Each assay was performed in triplicate. **P*<0.05, ***P*<0.01, and ****P*<0.001.

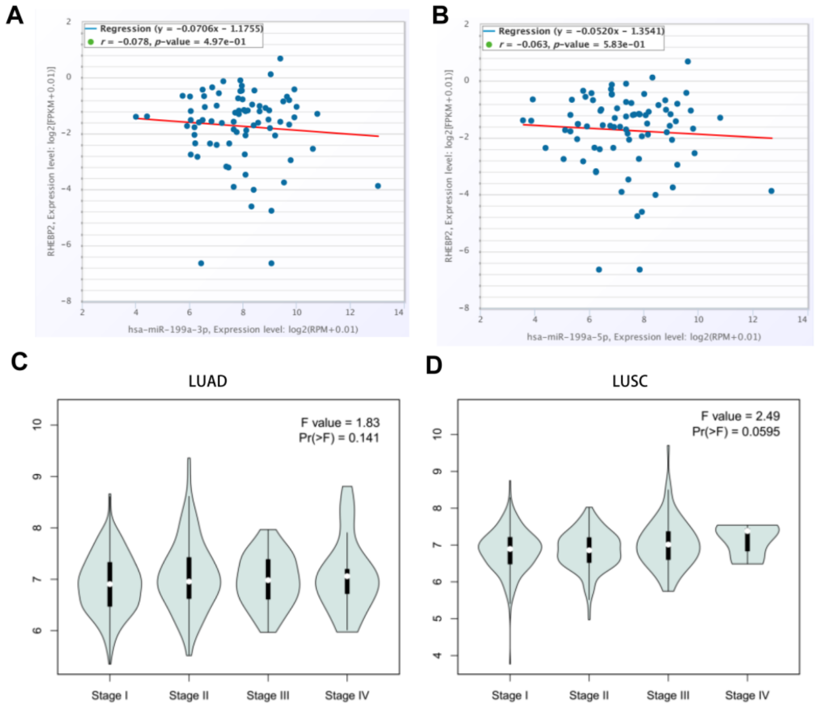

Figure Supplementary 4. (**A and B**) There was a negative correlation between miR-199a-3p/5p and Rheb, using GEPIA (http://gepia.cancer-pku.cn/index.html) analysis. (**C and D**) Rheb expression levels related with tumor stage in LUAD and LUSC tissue samples.

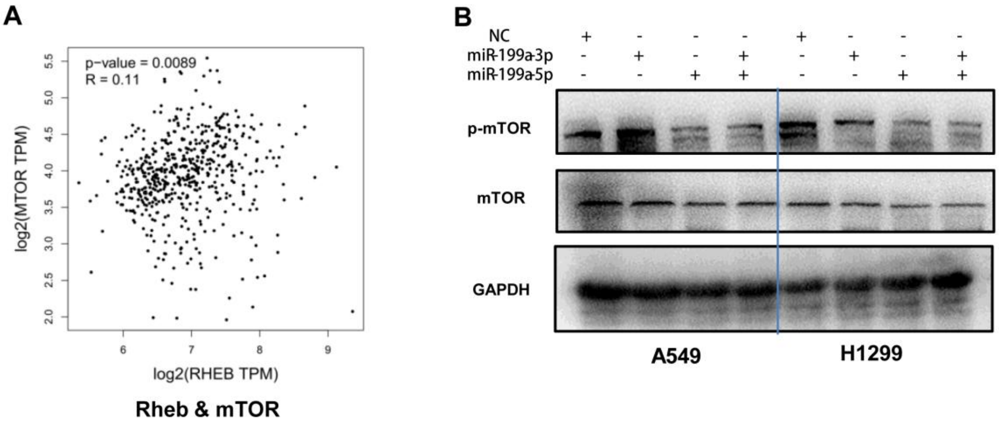

Figure Supplementary 5. (**A**) Rheb has a positive correlation with mTOR, through

ENCORI (http://starbase.sysu.edu.cn/index.php) database analysis. (**B**) revealed little change in was decreased In A549 and H1299 cells, the protein expression level of mTOR, p-mTOR after upexpression of miR-199a-3p and miR-199a-5p.

Supplementary Table 1 The clinical-pathological features of 74 cases NSCLC patients

No.	Gender	Age	Specimen Type	Histologic Type	pTNM
1	M	47	Pneumonectomy	Adenocarcinoma	T1bN0M0
2	M	78	Lobectomy	Adenocarcinoma	T2aN0M0
3	F	67	Pneumonectomy	Adenocarcinoma	T2N1M0
4	M	54	Lobectomy	Adenocarcinoma	T4N0M0
5	M	49	Lobectomy	Adenocarcinoma	T2bN2M0
6	F	66	Pneumonectomy	Adenocarcinoma	T2N2M0
7	M	67	Lobectomy	Adenocarcinoma	T2aN0M0
8	F	62	Lobectomy	Adenocarcinoma	T1aN0M0
9	F	75	Lobectomy	Adenocarcinoma	T2aN0M0
10	F	58	Lobectomy	Adenocarcinoma	T2aN1M0
11	F	65	Lobectomy	Adenocarcinoma	T2aN0M0
12	M	72	Lobectomy	Adenocarcinoma	T2aN0M0
13	M	64	Lobectomy	Adenocarcinoma	T1aN0M0
14	F	62	Lobectomy	Adenocarcinoma	T2aN0M0
15	M	65	Lobectomy	Adenocarcinoma	T4N2M0
16	F	55	Lobectomy	Adenocarcinoma	T2aN1M0
17	M	50	Lobectomy	Adenocarcinoma	T2aN0M0
18	F	67	Pneumonectomy	Adenocarcinoma	T2N1M0
19	M	67	Lobectomy	Adenocarcinoma	T2aN0M0
20	M	71	Lobectomy	Adenocarcinoma	T1bN0M0
21	F	64	Lobectomy	Adenocarcinoma	T2aN0M0
22	M	62	Lobectomy	Adenocarcinoma	T2aN0M0
23	M	58	Lobectomy	Adenocarcinoma	T2aN0M0

24	M	70	Lobectomy	Adenocarcinoma	T3N0M0
25	M	59	Lobectomy	Adenocarcinoma	T2aN0M1a
26	F	72	Lobectomy	Adenocarcinoma	T2aN0M0
27	M	51	Lobectomy	Adenocarcinoma	T1bN0M0
28	M	51	Lobectomy	Adenocarcinoma	T1aN0M0
29	F	55	Lobectomy	Adenocarcinoma	T1bN0M0
30	F	53	Lobectomy	Squamous cell carcinoma	T3N0M0
31	M	54	Lobectomy	Squamous cell carcinoma	T4N2M0
32	M	58	Lobectomy	Squamous cell carcinoma	T3N2M0
33	M	65	Lobectomy	Squamous cell carcinoma	T1bN0M0
34	M	65	Lobectomy	Squamous cell carcinoma	T2aN2M0
35	M	67	Lobectomy	Squamous cell carcinoma	T2aN1M0
36	M	67	Lobectomy	Squamous cell carcinoma	T2aN1M0
37	M	69	Lobectomy	Squamous cell carcinoma	T2aN0M0
38	M	71	Lobectomy	Squamous cell carcinoma	T2aN2M0
39	M	61	Lobectomy	Squamous cell carcinoma	T1bN0M0
40	M	67	Lobectomy	Adenocarcinoma	T2aN0M0
41	F	64	Lobectomy	Adenocarcinoma	T2aN2M0
42	M	60	Lobectomy	Adenocarcinoma	T1bN0M0
43	F	57	Lobectomy	Adenocarcinoma	T1bN0M0
44	M	65	Lobectomy	Combined Small Cell carcinoma	T2bN2M0
45	M	77	Lobectomy	Squamous cell carcinoma	T2aN0M0
46	M	71	Lobectomy	Adenocarcinoma	T1bN0M0
47	F	64	Lobectomy	Adenocarcinoma	T2aN0M0
48	F	55	Lobectomy	Adenocarcinoma	T1bN2M0
49	M	61	Lobectomy	Squamous cell carcinoma	T1bN0M0
50	M	62	Lobectomy	Adenocarcinoma	T2aN0M0
51	M	58	Lobectomy	Adenocarcinoma	T2aN0M0

52	F	60	Lobectomy	Adenocarcinoma	T1bN0M0
53	F	61	Lobectomy	Adenocarcinoma	T2aN1M0
54	F	67	Lobectomy	Adenocarcinoma	T2aN1M0
55	F	57	Lobectomy	Adenocarcinoma	T4N0M0
56	F	54	Lobectomy	Adenocarcinoma	T2aN3M0
57	F	66	Lobectomy	Adenocarcinoma	T1bN0M0
58	M	64	Lobectomy	Adenocarcinoma	T2bN2M0
59	M	49	Lobectomy	Adenocarcinoma	T1bN0M0
60	M	65	Lobectomy	Adenocarcinoma	T1bN0M0
61	F	59	Lobectomy	Adenocarcinoma	T2aN1M0
62	F	62	Lobectomy	Adenocarcinoma	T1bN0M0
63	F	45	Lobectomy	Adenocarcinoma	T2bN2M0
64	M	69	Lobectomy	Adenocarcinoma	T2bN2M0
65	M	65	Lobectomy	Adenocarcinoma	T2aN1M0
66	F	58	Lobectomy	Adenocarcinoma	T2aN1M0
67	M	59	Lobectomy	Adenocarcinoma	T2N1M0
68	M	60	Lobectomy	Squamous cell carcinoma	T1bN0M0
69	M	62	Lobectomy	Adenocarcinoma	T2aN1M0
70	F	55	Lobectomy	Squamous cell carcinoma	T2aN1M0
71	F	65	Lobectomy	Squamous cell carcinoma	T2aN2M0
72	F	48	Lobectomy	Adenocarcinoma	T2aN0M0
73	M	71	Lobectomy	Adenocarcinoma	T2aN2M0
74	F	55	Lobectomy	Adenocarcinoma	T2aN0M0

Supplementary Table 2: Primer sequences information table

Name	Sequence (5′–3′)
18S RNA (F)	CTCGCTTCGGCAGCACA
18S RNA (R)	GCCTCACTAAACCATCCAA
U6 snRNA (F)	CTCGCTTCGGCAGCACA
U6 snRNA (R)	AACGCTTCACGAATTTGCGT
miR-199a-3p qRT	ACAGTAGTCTGCACATTGGTTA
miR-199a-5p qRT	CCCAGTGTTCAGACTACCTGTTC
Rheb-3′-UTR (F)	GCTCTAGAGGGGAAAGTACAAATACC
Rheb-3′-UTR (R)	CGGAATTCATAATCTGAAGGGAGGG
3p-Rheb-3′-mUTR (F)	TGTTACTCAAGTATTAACTAGGCTTCAGTATA
3p-Rheb-3′-mUTR (R)	TTTTACTACGTATAACAATGAGTTCATAATTG
5p-Rheb-3′-mUTR (F)	CTGCTGCAAAGCCTGAGGTCTCAGCGAATA
5p-Rheb-3′-mUTR (R)	GCTTCTTCAGGTAGAATATATTCGCTGAGACCTCAG
Rheb qRT (F)	CTATCTTTCCTCAGACATACTCCA
Rheb qRT (R)	CACCATATCCAACAATTTGCCATG
shRheb-1(F)	CCGGGTTGGTTGGGAATAAGAAAGACTCGAGTCTTTCTTATT CCCAACCAACTTTTTG
shRheb-1(R)	AATTCAAAAAGTTGGTTGGGAATAAGAAAGACTCGAGTCTT TCTTATTCCCAACCAAC
shRheb-2(F)	CCGGGCAAGTCTTCATGCTCGGTGACTCGAGTCACCGAGCAT GAAGACTTGCTTTTTG
shRheb-2(R)	AATTCAAAAAGCAAGTCTTCATGCTCGGTGACTCGAGTCACC GAGCATGAAGACTTGC

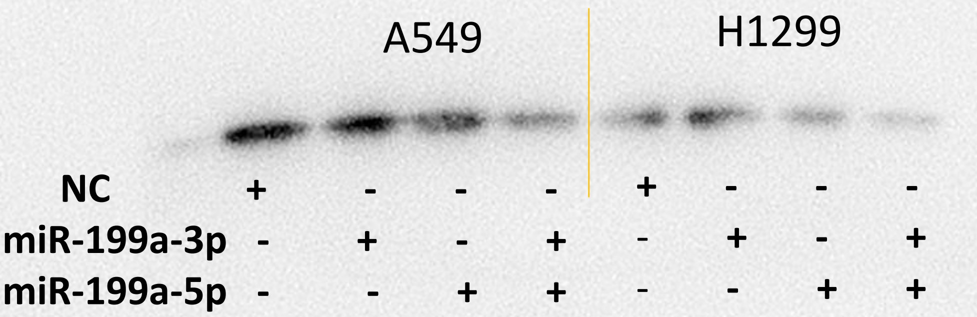

Original Figure 3M-Rheb-1

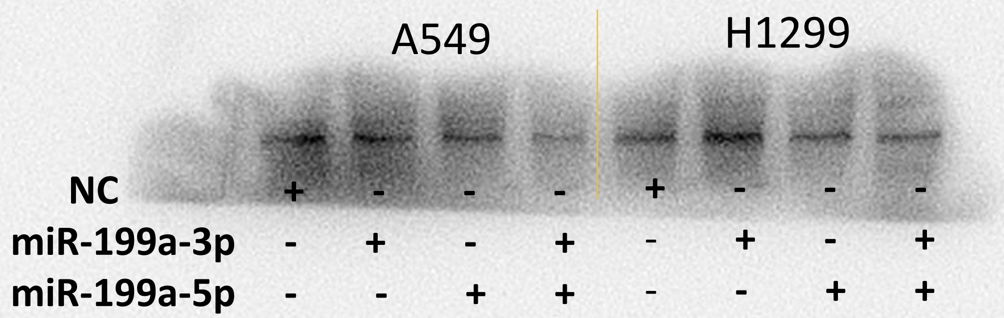

Original Figure 3M-Rheb-2

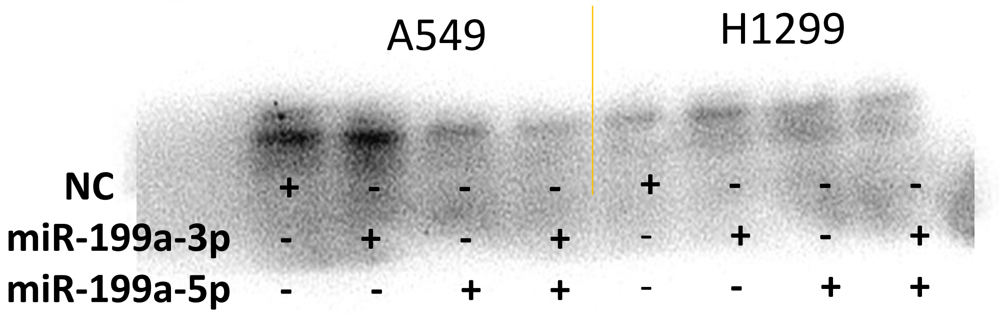

Original Figure 3M-Rheb-3

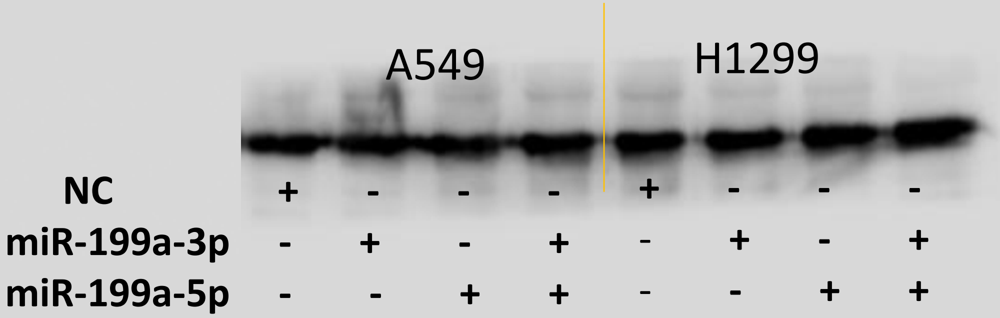

Original figure 3M-GAPDH-1

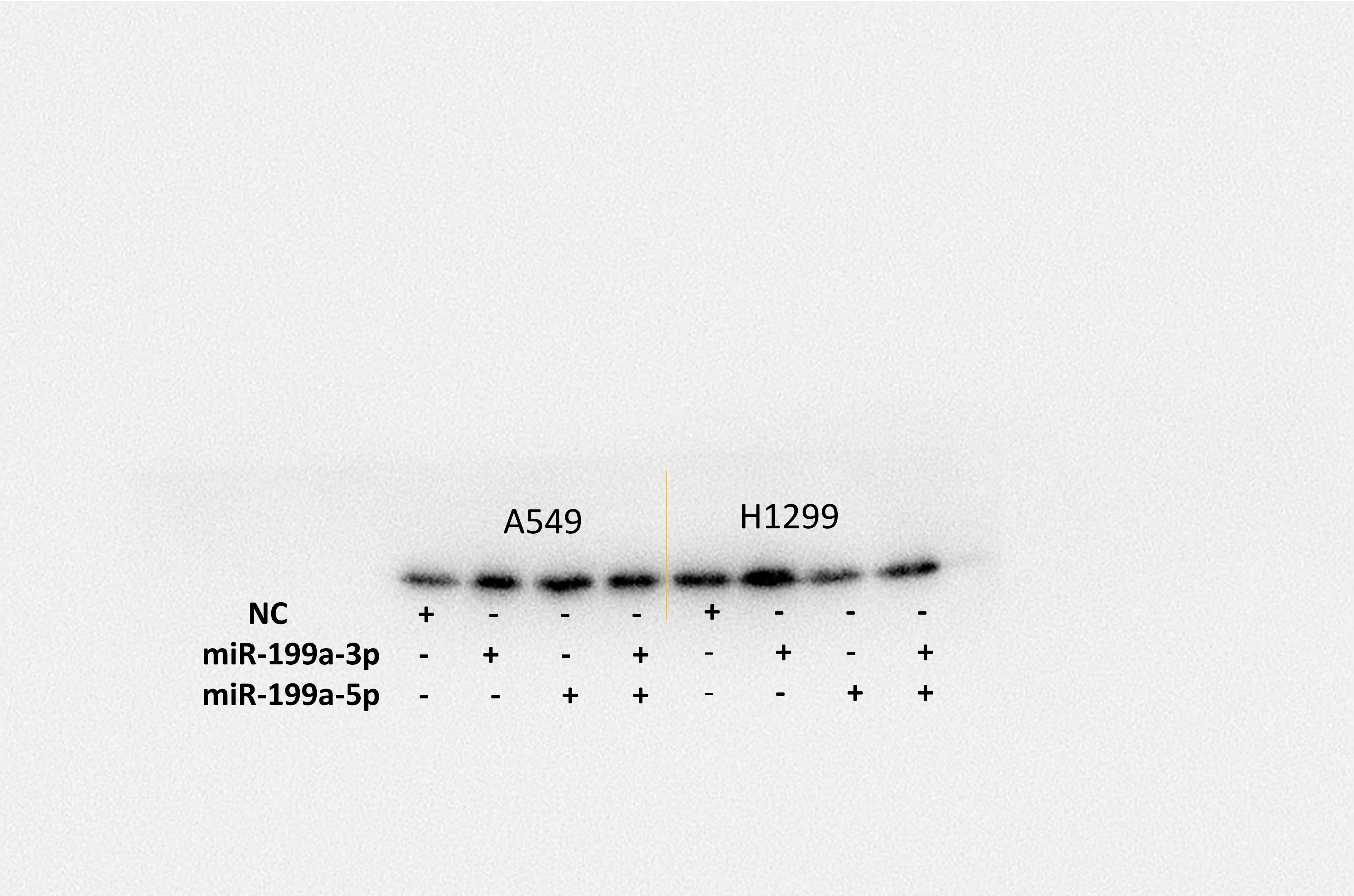

Original figure 3M-GAPDH-2

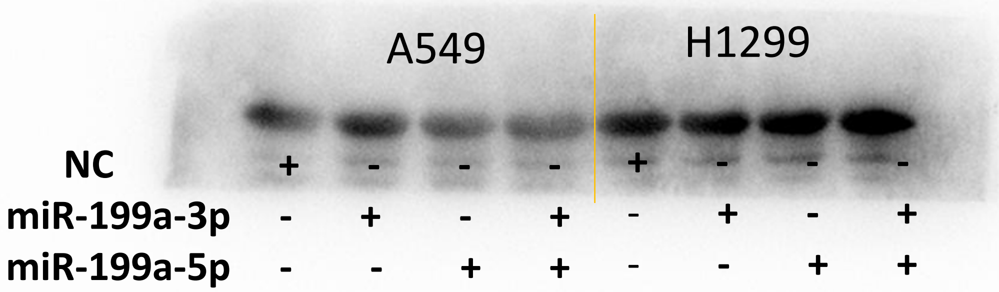

Original figure 3M-GAPDH-3

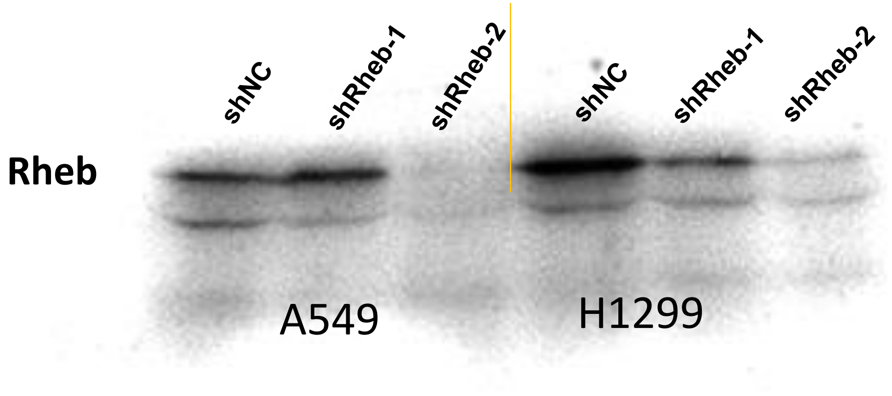

Original Figure 4B-Rheb
First

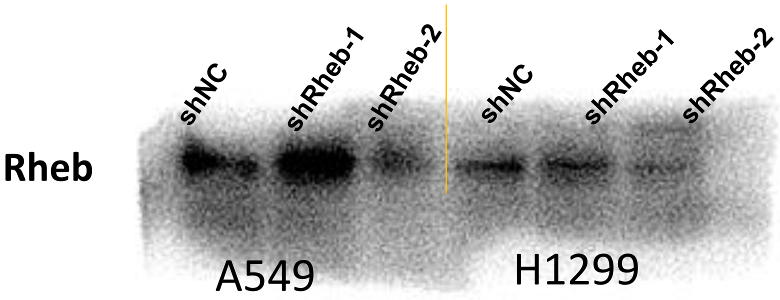

Original Figure 4B-Rheb
Second

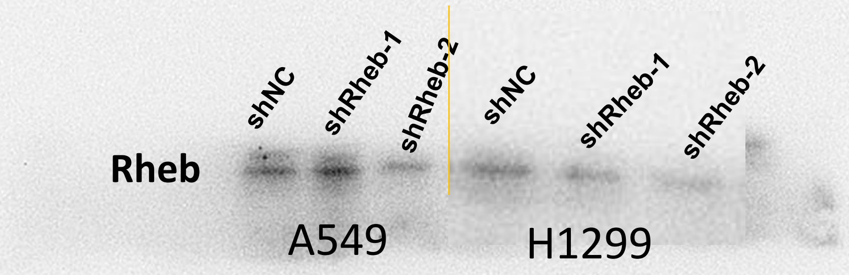

Original Figure 4B-Rheb
Third

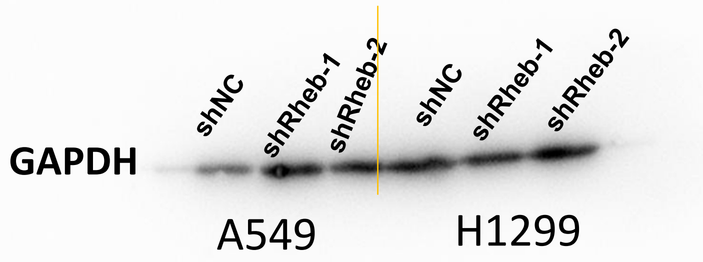

Original Figure 4B-GAPDH-1

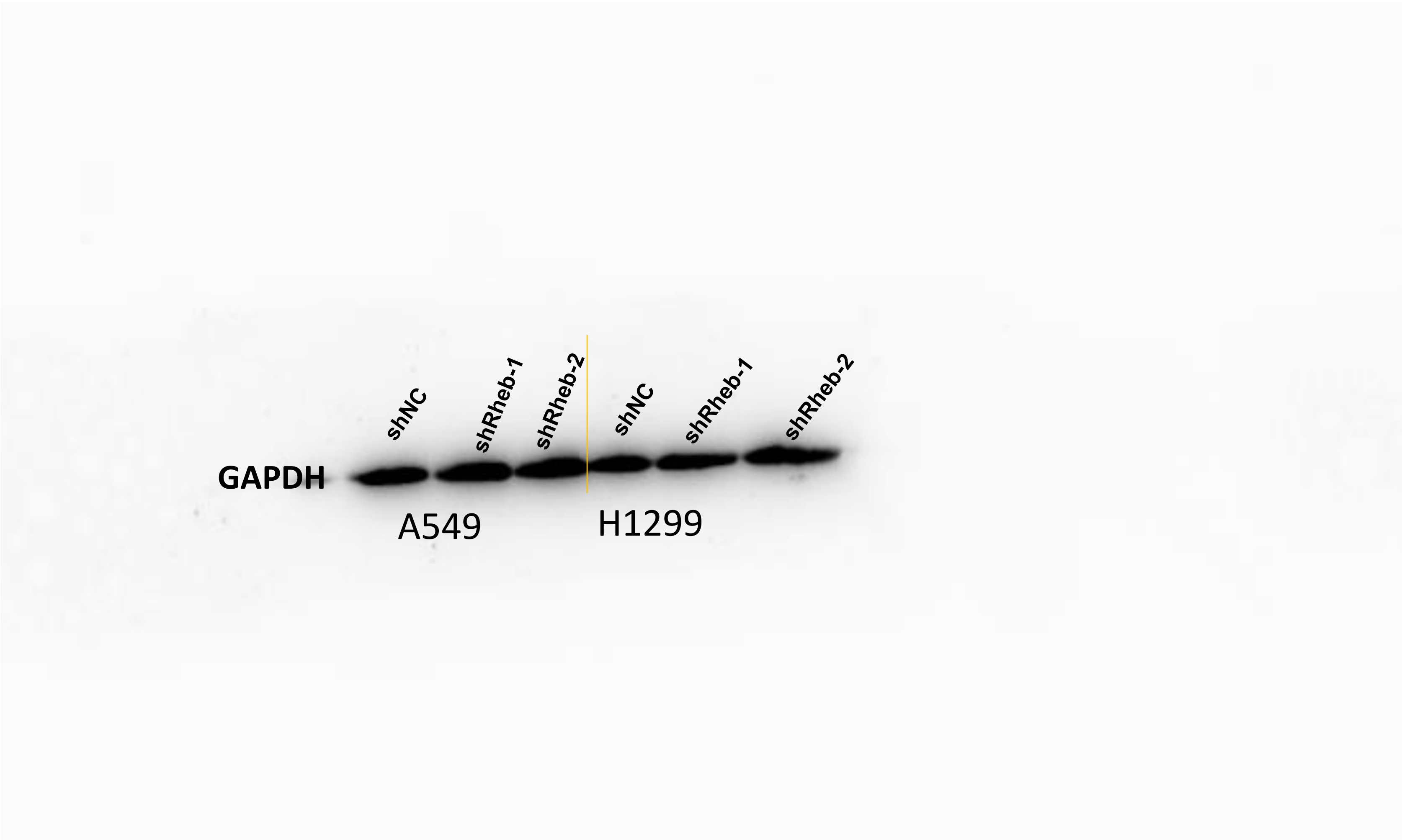

Original Figure 4B-GAPDH-2

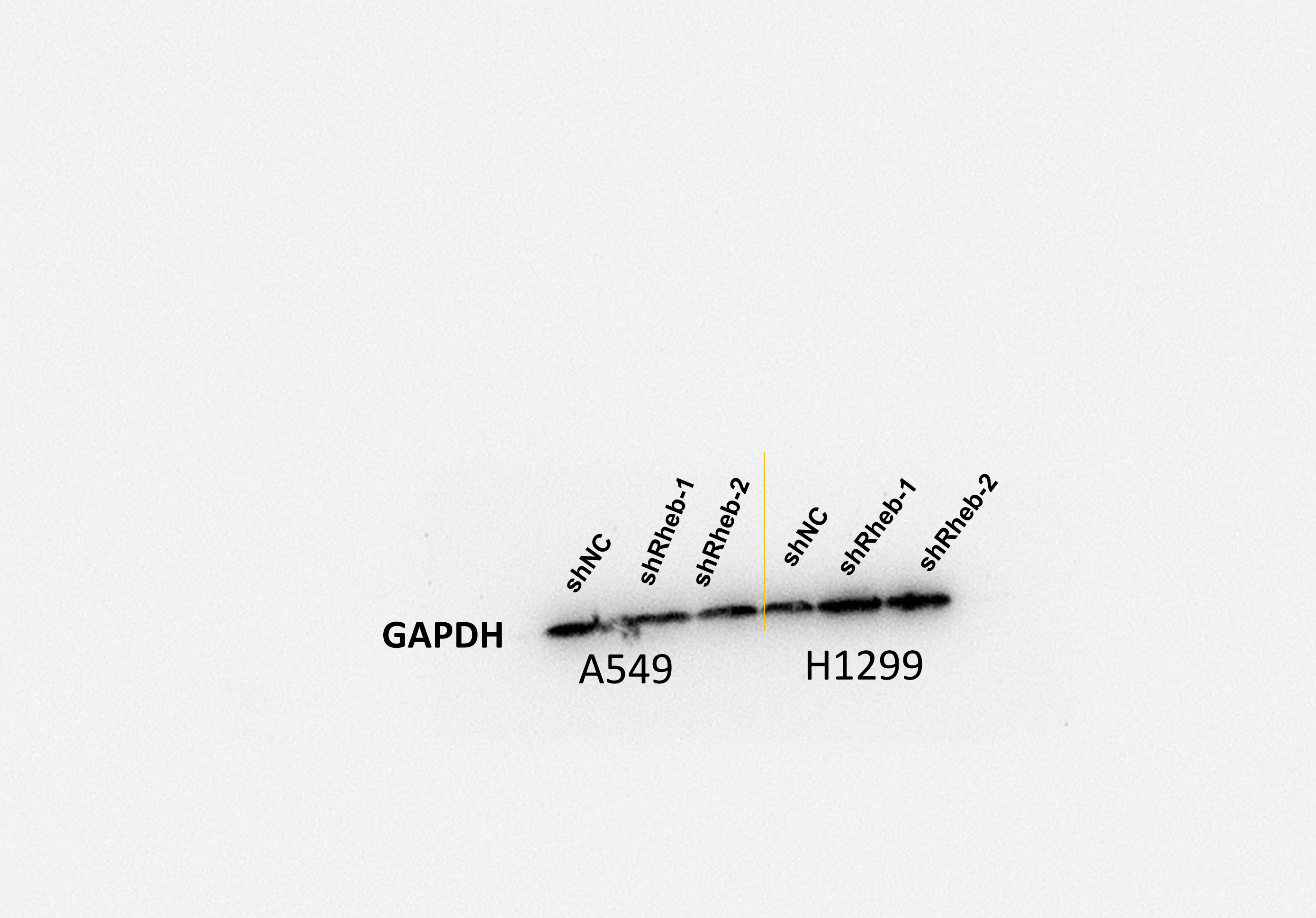

Original Figure 4B-GAPDH-3

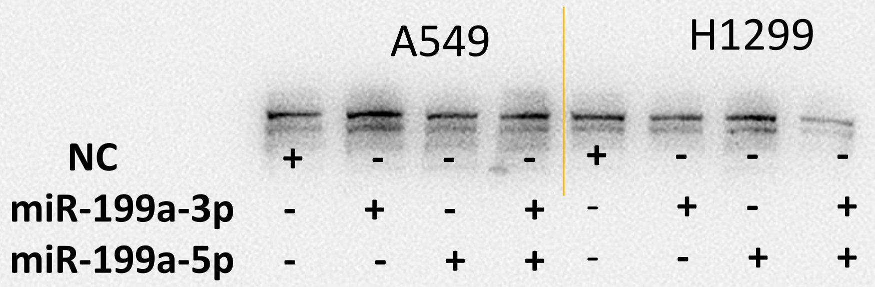

Original Supplementary Figure 5B-p-mTOR
First

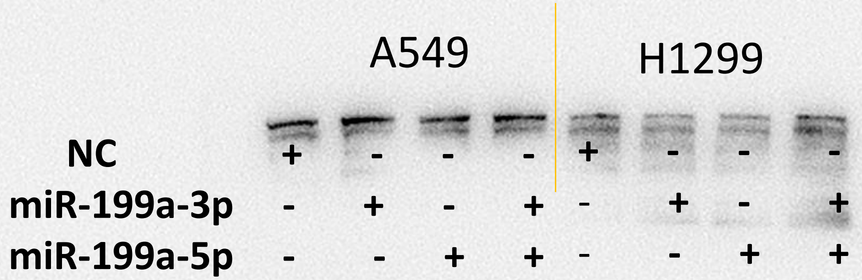

Original Supplementary Figure 5B-p-mTOR
Second

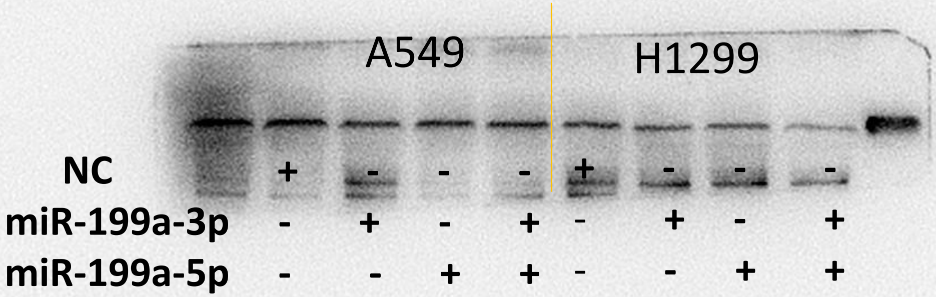

Original Supplementary Figure 5B-mTOR
First

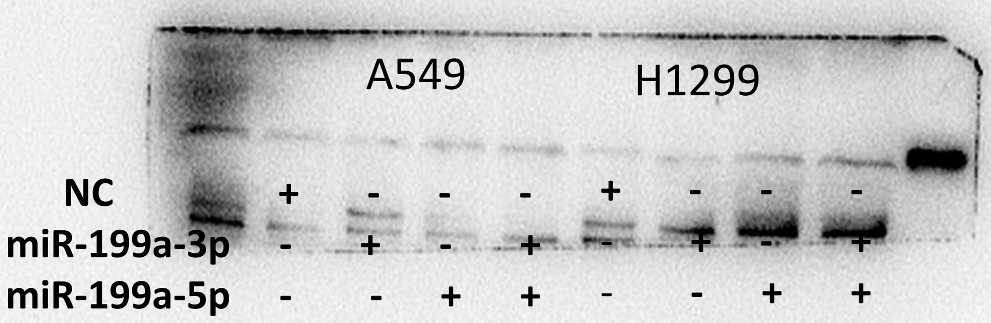

Original Supplementary Figure 5B-mTOR
Second

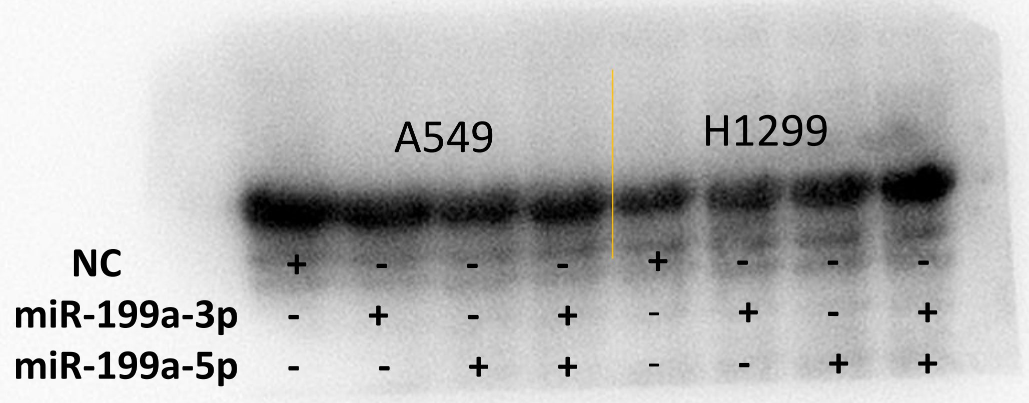

Original supplementary figure 5B-GAPDH-1

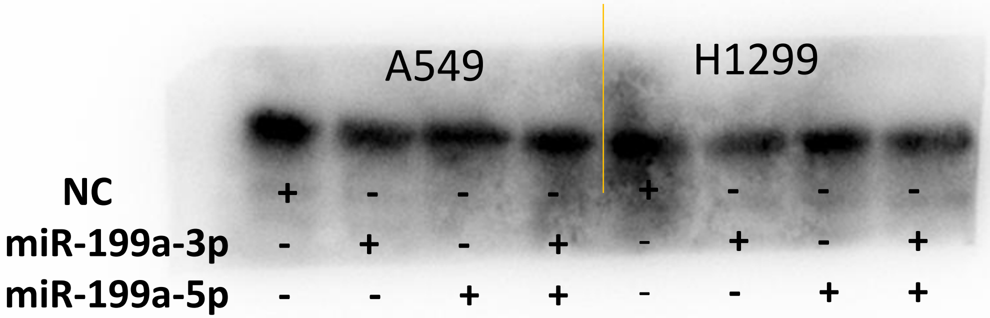

Original supplementary figure 5B-GAPDH-2
